# Investigating the interactive effects of habitat type and light intensity on rocky shores

**DOI:** 10.1007/s00442-024-05591-2

**Published:** 2024-07-24

**Authors:** Nina Schaefer, Katherine A. Dafforn, Emma L. Johnston, Graeme F. Clark, Mariana Mayer-Pinto

**Affiliations:** 1 Centre for Marine Science and Innovation, Evolution and Ecology Research Centre, School of Biological, Earth and Environmental Sciences, University of New South Wales, Sydney, NSW 2052 Australia; 2 School of Natural Sciences, Macquarie University, North Ryde, NSW 2109 Australia; 3 School of Life and Environmental Sciences, University of Sydney, Camperdown, NSW 2006 Australia

**Keywords:** Intertidal, Mobile species, Rock pools, Algae, Shading

## Abstract

Light availability and habitat complexity are two key drivers of community assembly. Urbanisation has been shown to affect both, with important consequences to ecological communities. On the intertidal, for instance, studies have shown that light intensity is greater on natural rocky shores than on less complex artificial habitats (seawalls), though different habitats can also experience similar light intensities, for example when shaded by urban structures. Understanding therefore how these factors individually, and combined, affect communities is important to understand the mechanisms driving changes in community structure, and consequently provide solutions to tackle the increasing homogenisation of habitats and lightscapes in urbanised spaces through smart infrastructure designs. Here, we assessed how different light levels affect the recruitment of communities in rock pools and on emergent rock on an intertidal rocky shore. We cleared 30 patches of emergent rock and 30 rock pools and manipulated light using shades with different light transmissions (full light, procedural control, 75%, 35%, and 15% light transmission, full shade) and assessed mobile and sessile communities monthly for 6 months. Effects of reducing light levels were generally stronger on rock than in pools. Fully shaded plots supported double the amount of mobile organisms than plots in full sunlight, in both habitats. Algal cover was higher in pools compared to rock, and at intermediate light levels, but effects varied with site. This study highlights the importance of variable light conditions and different habitats for rocky shore communities, which should be considered in future coastal developments to retain natural biodiversity.

**Supplementary Information:**

The online version contains supplementary material available at 10.1007/s00442-024-05591-2.

## Introduction

Sunlight affects the structure and functioning of ecological communities, including the primary productivity of terrestrial and aquatic plants (Duarte 1995; Fankhauser and Chory 1997), biotic interactions such as predation and competition (Bennie et al. 2014; Bolton et al. 2017; Duffy-Anderson and Able 2001; Gutman and Dayan 2005), species migrations (Moore et al. 2000; Ono and Simenstad 2014; Van Doren et al. 2017), and larval recruitment (Blockley and Chapman 2006). Additionally sunlight can, through associated infrared radiation (heat), affect bodily functions, for example, by increasing metabolic functions in ectotherms (Judd 1975) or causing heat stress when upper thermal limits are exceeded (Jurgens and Gaylord 2018, Seabra et al. 2011). The complex architecture of natural environments moderates light conditions through structural complexity (e.g., shaded microhabitats) and habitat-formers (e.g., vegetation) (Anthony and Hoegh-Guldberg 2003; Bertness et al. 1999; Fetcher 1984; Sebens 1991), which facilitate the development of a range of microclimates (Sebens 1991) that can buffer changes in natural light availability and associated stressors. Habitat complexity can thus indirectly affect ecological interactions and processes, with important implications for ecosystem functions and the services they underpin. While the influence of light on biotic interactions has often been considered, the interactive effects of light and habitat complexity on ecological communities have rarely been explored.

Changes in the light intensity and duration of exposure are likely to have important consequences to ecosystems, and the effects vary depending on the availability of habitat and refuges, as well as between functional groups. For example, autotroph organisms on land and in aquatic environments rely on light for photosynthesis and growth (Anthony and Fabricius 2000; Chazdon and Fetcher 1984), and thus tend to be less abundant in shaded areas (Blockley 2007; Miller and Etter 2008). In contrast, strong light and high UV levels may cause photoinhibition and photodamage, reducing growth and causing bleaching (Pessoa 2012; Powles 1984). Other non-autotroph organisms, such as marine sessile invertebrates, benefit from reduced competition from autotrophs under low-light levels and show increased abundance in shaded conditions (Glasby 1999; Miller and Etter 2008). Additionally, many invertebrate larvae are negatively phototactic (Thorson 1964) and settle into darker microhabitats like pits (Schaefer et al. 2023). The amount of light available may also influence the behaviour of mobile species. For instance, visual predators, such as marine and freshwater fish, rely on light for predation and avoid shaded surfaces (Benton et al. 2008; Duffy-Anderson and Able 2001).

Many mobile species are also reliant on shaded microhabitats that provide refuge/shelter from stressors associated with high light intensities (i.e., heat). Terrestrial ectotherms like lizards show behavioural adaptations to heat stress by seeking shaded microhabitats (e.g., crevices) to avoid lethal overheating at daytimes when solar radiation is strongest (López-Alcaide et al. 2017). Similarly, in coastal intertidal areas, mobile organisms move towards shaded crevices or rock pools that provide more stable conditions than exposed areas, where they are less thermally stressed and experience reduced water loss (Garrity 1984; Gray and Hodgson 2004; Metaxas and Scheibling 1993). Additionally, some algae are limited to water-retaining microhabitats like rock pools due to their susceptibility to desiccation as a result of sun exposure (Martone et al. 2010). Modifications of naturally variable light conditions to static conditions may therefore cause shifts in community structure and reduce overall diversity to a few dominant taxa. Organisms with narrow physiological tolerances may be excluded from land- and seascapes of light extremes where microhabitats that buffer light-related stressors are unavailable (Li et al. 2021; Scheffers et al. 2014).

In coastal urban areas, the construction of artificial structures along the coastline, such as overwater structures (e.g., bridges, wharves) and high-rise buildings, alters the natural light regime of large areas by shading nearby habitats, in some cases permanently (Munsch et al. 2017; Pardal‐Souza et al. 2017). In these areas, both above and under the waterline, constant shade faciliatates invertebrate-dominated communities (Blockley 2007). Similarly, vertical, homogeneous built infrastructure such as seawalls does not only lack protective microhabitats often found on natural shorelines, such as rock pools, crevices, and overhangs, (Chapman and Bulleri 2003), but also alter the light environmment experienced by organisms inhabiting these artificial habitats (Trethewy et al. 2023). For example, light intensity is greater on more complex natural rocky shores than on less complex seawalls (Trethewy et al. 2023) Intertidal hard substrates are arguably one of the most naturally stressful environments due to alterations of immersion and emersion with the tidal cycle. When emerged, intertidal organisms are directly exposed to atmospheric conditions and sunlight, which introduce a variety of potential stressors including UV light, heat, and desiccation (Raffaelli and Hawkins 2012). The lack of refugia (e.g., rock pools, crevices) on infrastructure often results in the low abundance and cover of mobile organisms and seaweeds (Chapman 2003; Chapman and Blockley 2009). These consequences can be either due to the reduced area availability, lack of physical physical protection against, e.g., predators, or due to interactions with other stressors, such as light and temperature, as, for example, submerged habitats, such as rock pools, can alleviate changes in light and temperature(Metaxas and Scheibling 1993). It is important therefore to understand how changes on light conditions and how different types (and complexity) of habitats can either exacerbate or ameliorate effects of stressors and the consequences to intertidal communities.

Increasingly, ecological principles are being incorporated into designs of infreastructure such as seawalls and breakwaters, to improve their ecological value, a process known as ‘ecological engineering’ (Bergen et al. 2001). This includes modifications of built infrastructure to increase complexity, for example through the addition of artificial rock pools or shaded habitats like crevices (Browne and Chapman 2011; Chapman and Blockley 2009; Moreira et al. 2007; Morris et al. 2018a), to reduce permanent shading from overwater structures through installations of light-transmissive surfaces (Blanton et al. 2002; Gayaldo and Nelson 2006; Morris et al. 2018b; Munsch et al. 2017), or both (Bilkovic et al. 2017; Dyson and Yocom 2015; Sawyer et al. 2020). Understanding the ecological mechanisms underpinning changes in intertidal communities affected by different levels of shading and complexity can help to optimise coastal development and minimise subsequent ecological impacts.

Here, light intensity was manipulated in Austral summer and autumn using artificial shades (75%, 35%, and 15% of natural light and full shade) to test the ecological responses of intertidal communities on rock pools and emergent rock compared to those under natural light conditions. This season was chosen at it is peak recruitment and growth time for algae (Underwood and Anderson 1994) and also represents the season of greatest thermal stress for intertidal organisms. A gradient of light intensities was used to look for thresholds of change from algal-dominated to invertebrate-dominated communities. The development of algal communities (measured as percentage cover) was expected to achieve greater cover under treatments of high light transmission, but that treatments of low-light transmission would have greater cover of sessile invertebrates and greater abundances of mobile taxa. Greater cover of sessile organisms and higher abundances of mobile taxa was expected in rock pools due to more stable thermal conditions and constant submersion in this habitat. Thus, a greater effect of shading treatments was expected on rock platforms.

## Materials and methods

### Study area

**Fig. 1:**
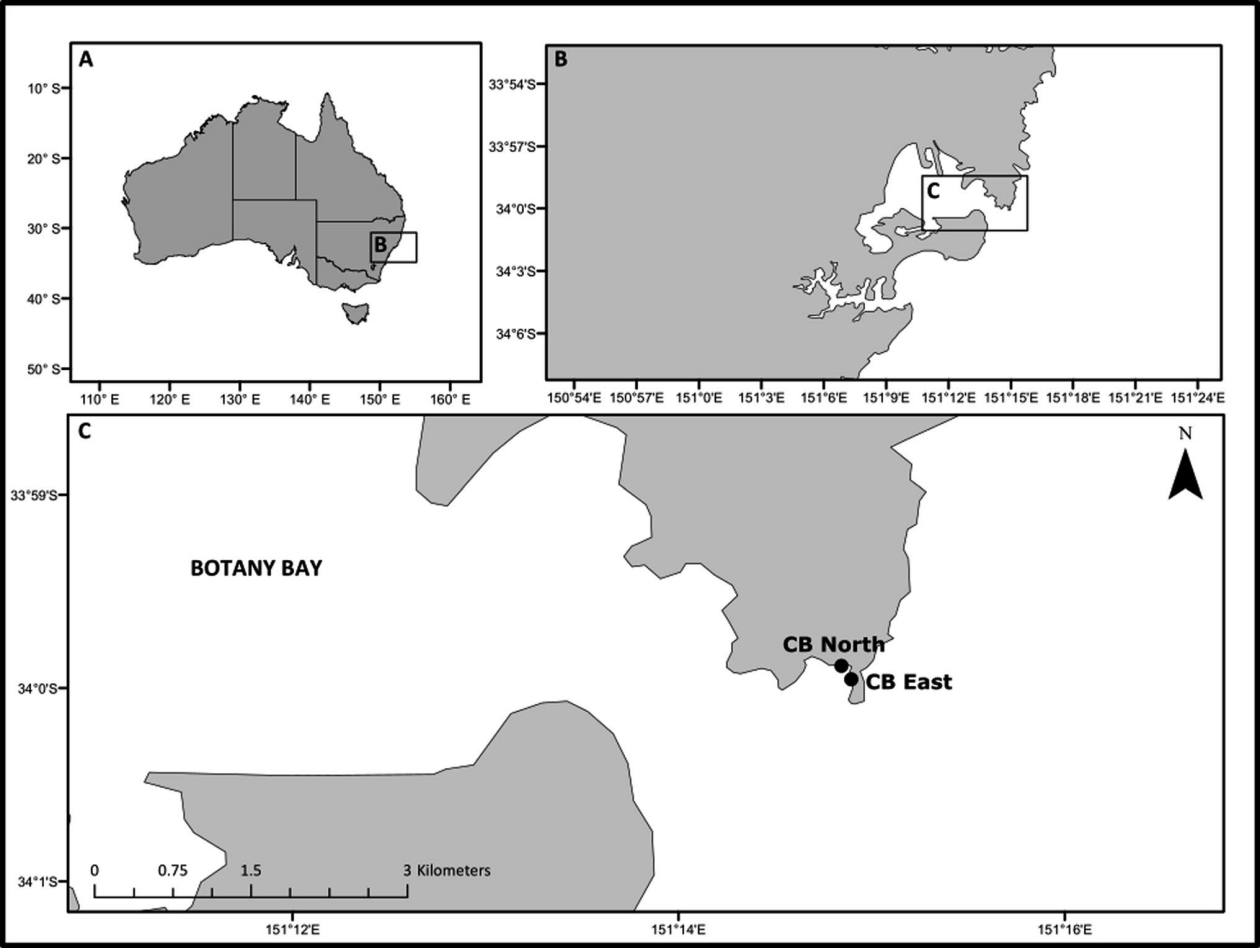
Map of Botany Bay, NSW, Australia showing the location of the two experimental sites. *CB* Cape Banks

The experiment was done at two sites (Cape Banks North and East) in the Cape Banks (CB) Scientific Marine Research Area on the northern headland of the Botany Bay National Park, New South Wales, Australia between 4 Dec 2016 and 5 Jun 2017 (Fig. 1). Sites were approximately 175 m apart. CB North was a sheltered rocky platform on the low intertidal, whereas CB East was an exposed site in the mid intertidal.

Each site had 45 artificially constructed cylindrical rock pools of 5 cm depth and 15 cm diameter. These were constructed in 1986 by drilling diamond-corers into the rock to create depressions of consistent depth and width (Underwood and Skilleter 1996). Pools at each site were approx. 0.3–1 m apart from one another. The intertidal communities around the Cape Banks area generally display distribution patterns similar to other rocky shores in NSW (SPCC1981; Underwood et al. 1983). Sea squirts (*Pyura*), macroalgae, and polychaetes (*Galeolaria caespitosa*) cover the lower levels of the shore, whereas mid intertidal levels mainly comprise of encrusting algae, gastropods, and barnacles. Periwinkles occupy the high levels on the shore (Denley and Underwood 1979; Underwood and Chapman 1996).

### Experimental set-up

Prior to the start of the experiment, we haphazardly chose 30 rock pools (from the available 45) and 30 patches (20 × 20 cm) of emergent rock at each site. We carefully cleared surfaces of biota using hammer and chisel and then scrubbed them with a wire brush and bleach. After this, we took a photo of each experimental plot directly from above to quantify any residual biota (i.e., residue of encrusting algae, hereafter referred to as ‘residual cover’) (performed on 4 Dec 2016).

**Fig. 2:**
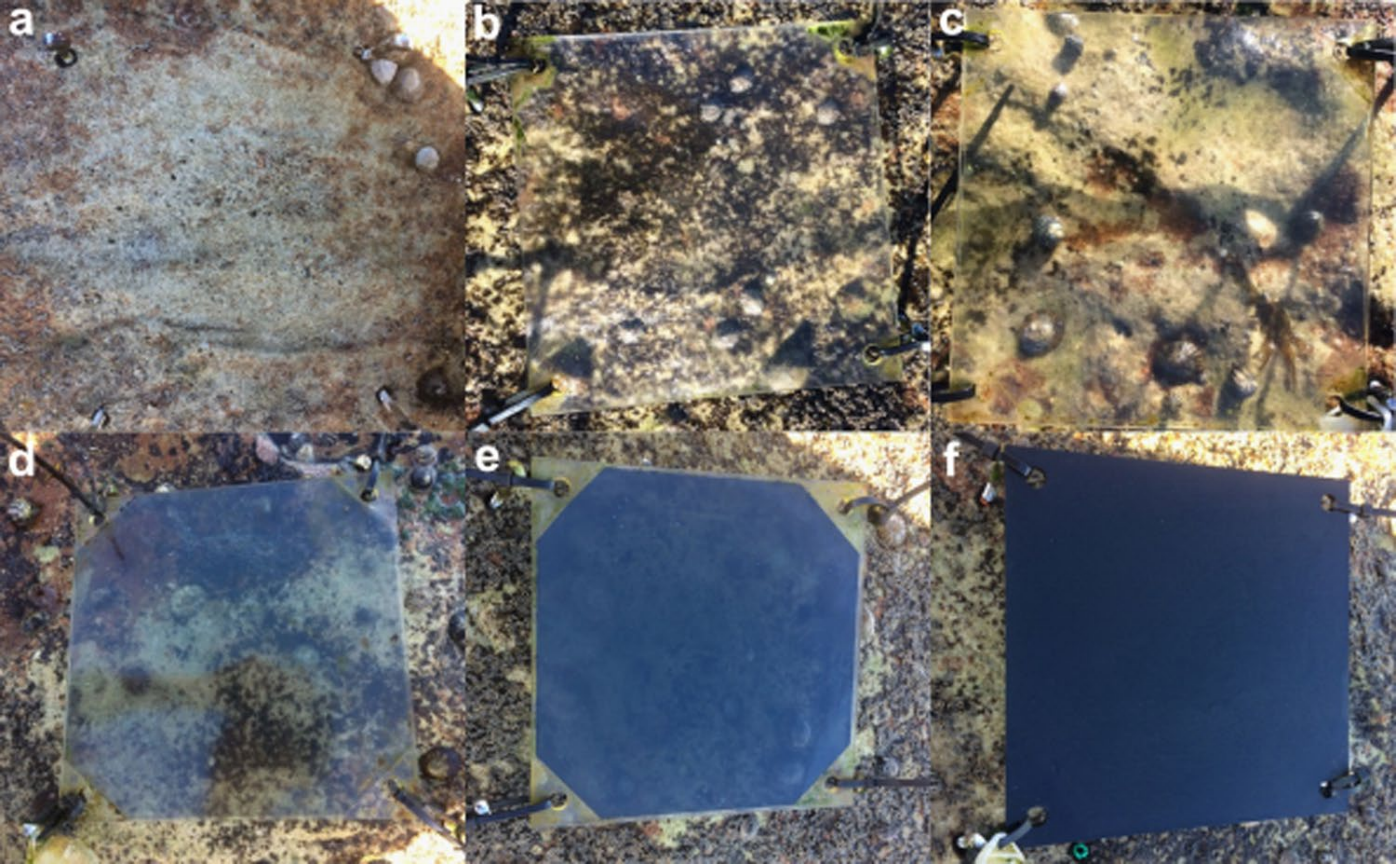
Light treatments used in the experiment. **a** Full light—no plate, **b** procedural control—clear plate no UV transmission, **c** 75% transmission no UV, **d** 35% transmission no UV, **e** 15% transmission no UV, and **f** full shade no UV

We randomly assigned experimental pools and patches (hereafter “plots”) to one of six light treatments: 0% (full shade), 15%, 35%, 75%, and 100% (full light) light transmission, and a procedural control (Fig. 2). Specifically, 40 experimental plots (20 rock pools and 20 emergent rock) were shaded with 20 × 20 cm, 4.5 mm-thick acrylic plates that were covered with tinted film to simulate the different light intensities while also inhibiting UV transmission (*n* = 5 replicates for each treatment by habitat type) (Table S1). Plates were attached ~ 1.5–4.5 cm above the plots with 4 stainless steel lag eye screws (M6 × 60) positioned around the rock pools/emergent rock (Fig. 2). To test for potential effects of the plates, we used a procedural control consisting of a clear acrylic plate over 10 experimental plots (5 rock pools and 5 emergent rock). We left the remaining 10 experimental plots for the ‘full light’ treatments (i.e., 100% light) uncovered.

We visited experimental sites every 1–2 weeks to remove any algal growth and salt accumulation on plates, and for the replacement of any lost plates. When plates were found missing during sampling, we did not assess mobile taxa at that sampling time as they were exposed to full sunlight for at least a full day. We replaced missing plates immediately, and mobile sampling continued in subsequent visits. Some tags and plates went missing simultaneously and we could not identify some plots which resulted in variable (*n* = 2–5) numbers of replicates between sampling times (Table S2).

### Light and temperature measurements

To measure the efficacy of treatments, and because changes in light is usually associated with changes in temperature, we took light and temperature measurements in each experimental plot using an HOBO temperature and light data logger, every second for 20 s, at each sampling time, placing it on emergent rock (measuring air temperature) or holding it at the bottom of the pool (measuring water temperature). We subsequently averaged the measurements across the 20 s to get one measurement per experimental plot per sampling time. To determine effects of shades on UV transmission, we measured UVA and UVB light once at the beginning of the experiment (12 Dec 2016) during a low tide with a light metre by placing it under each experimental plot. Measurements were noted down once values had stabilised (usually within a minute).

### Assessment of mobile and sessile colonisation

We assessed colonisation by mobile and sessile organisms in each experimental plot (i.e., rock pool or emergent rock) monthly for 6 months (sampling dates: 11 Jan, 9 Feb, 12 Mar, 6 Apr, 11 May, 5 Jun 2017). For communities within rock pools, we assessed only the base of the pool, so substrate orientation was consistent with the emergent rock (horizontal). We identified and counted all mobile organisms to the lowest possible taxonomic level. To assess colonisation of sessile organisms, we took one photo of each experimental plot (20 × 20 cm). At the laboratory, we estimated percentage cover of organisms in each experimental plot by placing a grid over each photograph. We used a round grid with 37 regularly spaced intersections to estimate cover of sessile taxa in rock pools and a square grid with 25 intersections for estimates on emergent rock. We used different numbers of intersections to allow for regular spacing to fit each shape. As the cleared intertidal area covered by the shades had no clear borders when the shade was removed for the photo, approximately 2.5 cm of the outer area between the screws was not assessed for sessile colonisation (Fig. S1). As such, we assessed an area of 225 cm^2^ and 176.71 cm^2^ for pools and emergent rock, respectively. When identification was not possible (e.g., individual was mainly covered by algae from the pool walls), we classified them as ‘unknown’. Sessile organisms were identified to genus level or morphospecies. Turfing algae represent several types of micro- and macroalgae which share an extensive low-lying morphology (Connell 2014). We classified shell fragments or small pebbles retained in the rock pool as ‘debris’.

### Statistical analyses

#### Light and temperature measurements

To investigate potential differences among light treatments and the effectiveness of treatments over time, we used linear mixed models in the R package ‘nlme’ (Pinheiro et al. 2017). The full model included the time (i.e., sampling 1–5) as a covariate, and an interaction between the categorical fixed factors treatment (full light, procedural control; 75% light transmission, 35% light transmission, 15% light transmission, full shade) and habitat (i.e., pool, rock). When the interaction was non-significant, an additive model was used. We included each replicate as a random factor, nested within site, to account for repeated sampling over time. We obtained p values using the Anova function in the R package ‘car’ (Fox et al. 2012). We used Tukey’s post hoc test in the R package ‘emmeans’ (Lenth et al. 2018) to test for differences among treatments. As overdispersion was present in the light data, we log-transformed them. We pooled data from all sites for analyses.

#### Biota

To investigate how mobile taxon richness, mobile abundances and sessile cover varied among treatments over time, we used generalised linear mixed models (GLMMs) in the package ‘glmmTMB’ (Magnusson et al. 2017). For analyses of mobile taxon richness and abundance, we included an interaction between the categorical fixed factors treatment, sampling time (i.e., sampling 1–6), and habitat (i.e., pool and emergent rock) in the model. We included each replicate as a random factor to account for repeated sampling over time. Sites were analysed separately due to differences in intertidal height and exposure. We obtained p values using the Anova function in the R package ‘car’ (Fox et al. 2012).

We explored significant differences between full light and other light treatments (Dunnett’s post hoc test) for each sampling time using contrasts within the R package ‘emmeans’ (Lenth et al. 2018). Similarly, we compared differences in ratios (i.e., effect sizes) between full light and other light treatments between habitats using contrasts within the R package ‘emmeans’ (Lenth et al. 2018). We compared ratios of abundances of mobile organisms between the full light and other treatments among habitats to standardise for different areas assessed per habitat. We adjusted p values using ‘holm’ when time was significant, and contrasts were required for multiple sampling times. Due to low cover of sessile organisms other than algae, statistical analyses could only be performed for algae. For algal cover, we only assessed the final timepoint (i.e., sampling time), and we therefore excluded time from the model. We used a Poisson distribution for models with taxon richness as the response, a negative binomial distribution (nbinom2) for mobile species abundances, and a binomial distribution for percentage cover of sessile taxa (live organisms only).

Differences in sessile taxon richness were not analysed statistically due to the low number of sessile taxa in the plots and limited taxonomic resolution of turfing algal species.

## Results

### Light transmission and temperature

**Fig. 3:**
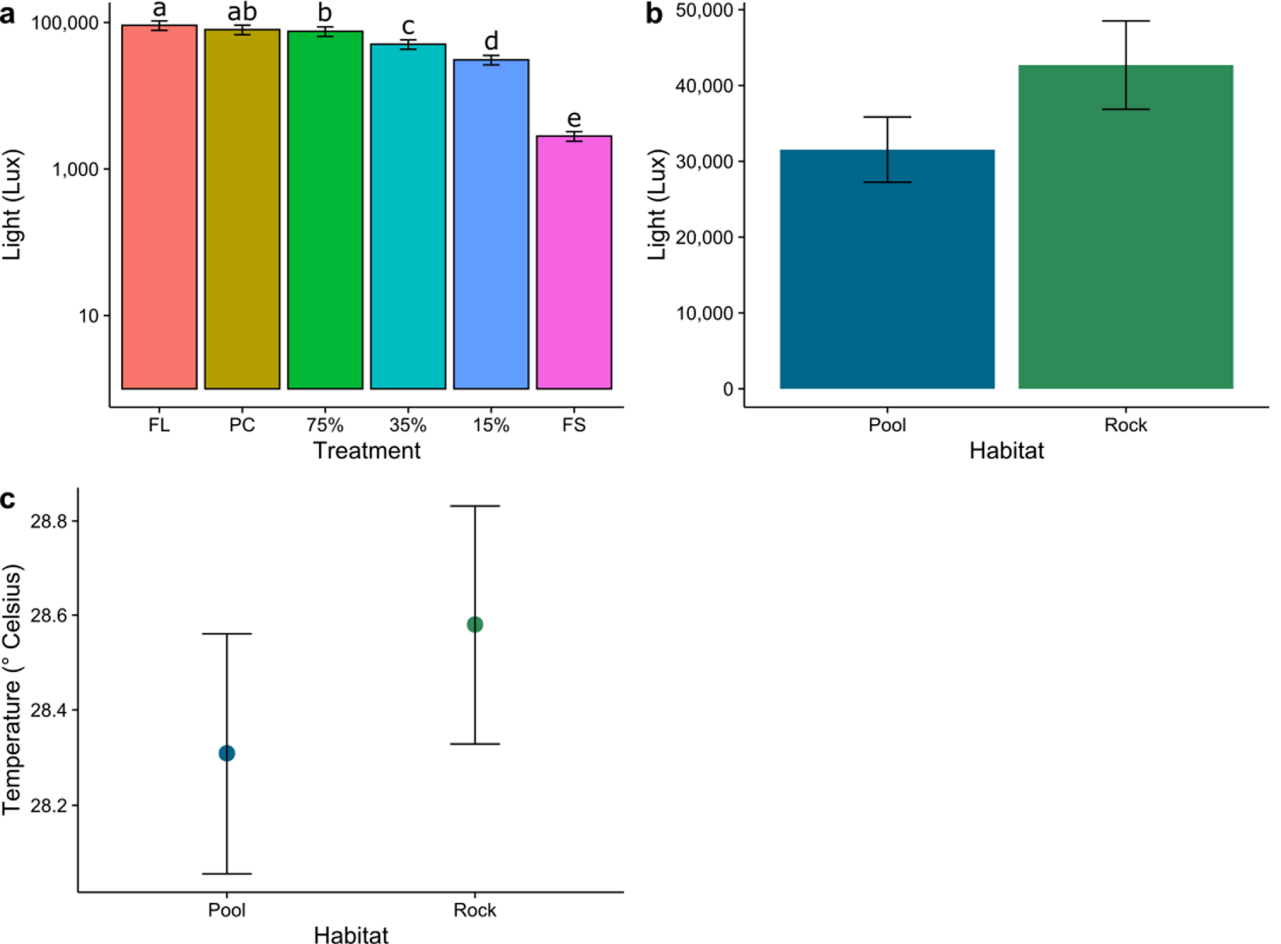
Light levels (Lux) for each **a** treatment and **b** habitat and **c** temperature (^o^ Celsius) for each habitat. Data are displayed on a log-scale. *FL* Full light, *PC*  procedural control, 75% = 75% light transmission, 35% = 35% light transmission, 15% = 15% light transmission, FS = full shade. Error bars are model predicted means and standard errors. Different letters indicate significant differences among light treatments. Light/temperature measurements are averaged across time and (**a**) habitats (*N* = 100 per treatment) and (**b** + **c**) treatments (N = 300 per habitat). Note the different scales

Experimental shades successfully created a gradient in the amount of light reaching the plots (Fig. 3a; Table S3). Light transmission to fully shaded (black plates), 15%, 35%, and 75% light transmission plots was significantly reduced compared to full light plots (no plates) (Table S3). Under the fully shaded treatments, ~ 3% of natural light was transmitted (Fig. 3a). Under the 15% and 35% light transmission plots, 34% and 55% of natural light were transmitted, respectively (Fig. 3a). Light transmission did not differ between the plots receiving full light and the procedural control plots (clear plates), and between the procedural controls and 75% light transmission plots (Fig. 3a; Table S4). Light intensity reaching the bottom of the pools was significantly lower than light intensities on rock, likely caused by light attenuation in the water (Fig. 3b). All plates inhibited the transmission of UV (Fig. S2). Temperature was also significantly lower in the pools than on emergent rock, whereas no differences between light treatments were found (Fig. 3c, Fig. S3; Table S5).

**Table 1:** Analysis of deviance table (type III Wald Chi-square tests) for mobile taxon richness, mobile abundance, and algal cover at CB North

Mobile richness	Chisq	Df	Pr(> Chisq)
(Intercept)	59.0536	1	1.534E-14***
Habitat	9.6960	1	0.001847*
Treatment	0.6593	5	0.9851280
Time	1.0819	5	0.9556960
Habitat:treatment	15.4773	5	0.008506*
Habitat:time	9.1516	5	0.1031680
Treatment:time	5.0126	25	0.9999940
Habitat:treatment:time	16.5040	25	0.8989920

Mobile abundance	Chisq	Df	Pr(> Chisq)
(Intercept)	171.9931	1	< 2.2E-16***
Habitat	24.7733	1	6.448E-07***
Treatment	11.4197	5	0.0436651*
Time	6.7536	5	0.2396217
Habitat:treatment	31.2704	5	8.283E-06***
Habitat:time	15.8284	5	0.0073515**
Treatment:time	19.4443	25	0.7753327
Habitat:treatment:time	54.4061	25	0.0005884***

Algal cover	Chisq	Df	Pr(> Chisq)
(Intercept)	4.6189	1	0.03162*
Treatment	9.3670	5	0.09529
Habitat	4.7387	1	0.02949*
Treatment:habitat	5.1003	5	0.40376

For algal cover, only the final timepoint (i.e., sampling 6) was assessed, and therefore, time was excluded from the model

Across the duration of the experiment, temperatures were highest (> 30 °C) during the first two sampling times (in January and February), and subsequently decreased to the mid (samplings in March and April) and low 20s (samplings in May and June) (Table S5).

#### Biota

We found a total of 29 mobile and 12 live sessile taxa (8 algae and 4 invertebrates) across all treatments, sampling times and sites (Table S6). Overall, plots at CB North supported higher abundances of mobile organisms and greater algal cover than plots at CB East. Communities at CB North were dominated by the grazing gastropod *Austrocochlea* spp. and the predatory gastropod *Tenguella marginalba*, whereas communities at CB East were dominated by the grazing gastropod *Nerita melanotragus* (Fig. S3 + S4). The most abundant sessile taxa in pools were the brown encrusting algae *Ralfsia* spp., followed by turfing algae and the green foliose algae *Ulva* spp (Fig. S6 + S7). Among sessile organisms, only algae (mainly *Ralfsia* spp.) recruited on emergent rock plots (Fig. S8).

#### Cape Banks North (CB North)

**Fig. 4:**
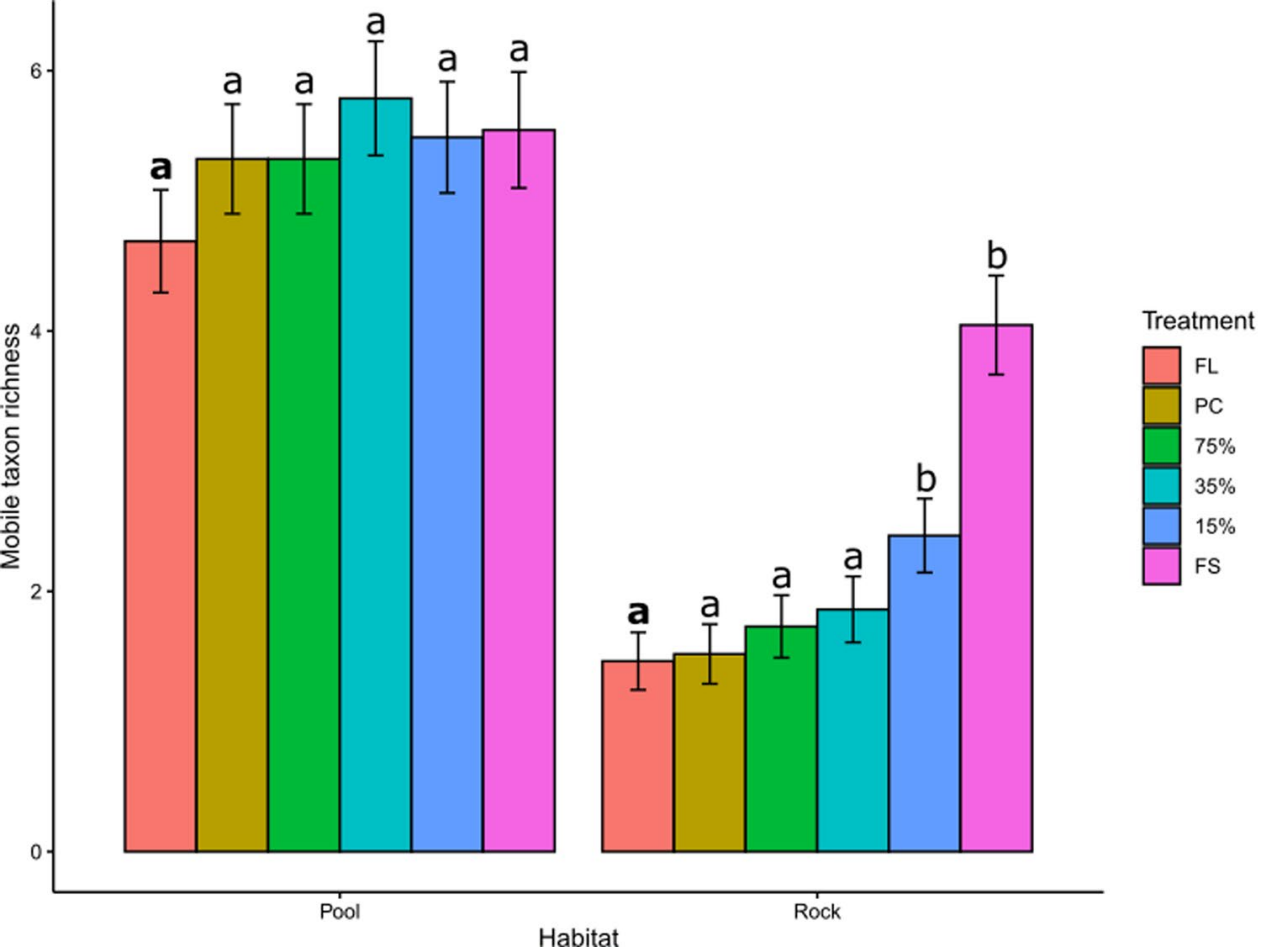
Mobile taxon richness for each treatment within pools and on emergent rock at CB North, averaged across time. *FL* full light, *PC* procedural control, 75% = 75% light transmission, 35% = 35% light transmission, 15% = 15% light transmission, FS = Full shade (*N* = 28–29 per habitat, see Table S2). Error bars are model predicted means and standard errors. Different letters indicate significant differences among the full light treatment (highlighted in bold) and other light treatments

Mobile taxon richness varied significantly among treatments and between habitats, but not through time (Table 2). Pairwise comparisons among light treatments within each habitat revealed that there was no difference in taxon richness across treatments within pools, but that taxon richness was significantly higher on emergent rock plots under 15% light transmission and full shade than plots exposed to full sunlight (Fig. 4, see supplementary material 2 for results for model results).

**Table 2:** Analysis of deviance table (type III Wald Chi-square tests) for mobile taxon richness, mobile abundance, and algal cover at site 1

Mobile richness	Chisq	Df	Pr(> Chisq)
(Intercept)	10.8625	1	0.0009813***
Habitat	6.6002	1	0.0101969*
Treatment	4.8258	5	0.4375026
Time	2.0400	5	0.8435851
Habitat:treatment	8.4536	5	0.1329453
Habitat:time	10.0822	5	0.0729399
Treatment:time	8.7804	25	0.9988622
Habitat:treatment:time	20.5383	25	0.7180497

Mobile abundance	Chisq	Df	Pr(> Chisq)
(Intercept)	116.7600	1	< 2.2E-16***
Habitat	41.3820	1	1.252E-10***
Treatment	26.7440	5	6.397E-05***
Time	12.4330	5	0.0293144*
Habitat:treatment	22.7030	5	0.0003847***
Habitat:time	37.7720	5	4.193E-07***
Treatment:time	38.7510	25	0.0390129*
Habitat:treatment:time	66.5560	25	1.233E-05***

Algal cover	Chisq	Df	Pr(> Chisq)
(Intercept)	1.8952	1	0.168621
Treatment	15.5421	5	0.008281**
Habitat	0.0000	1	0.997791
Treatment:habitat	5.1561	5	0.397124

For algal cover, only the final timepoint (i.e., sampling 6) was assessed, and therefore, time was excluded from the model

Comparisons of the ratio (i.e., the effect size) between the different light treatments and the full light treatment among the two habitats revealed that the reduced light levels did not have different effects on taxon richness when comparing habitats, except when full light and full shade were compared (Fig. S9, see supplementary material 2 for results for model results). Reducing the light levels from 100% (full sunlight) to 0% (full shade) increased mobile taxon richness by more than double on emergent rock than in pools (Fig. S10).

**Fig. 5:**
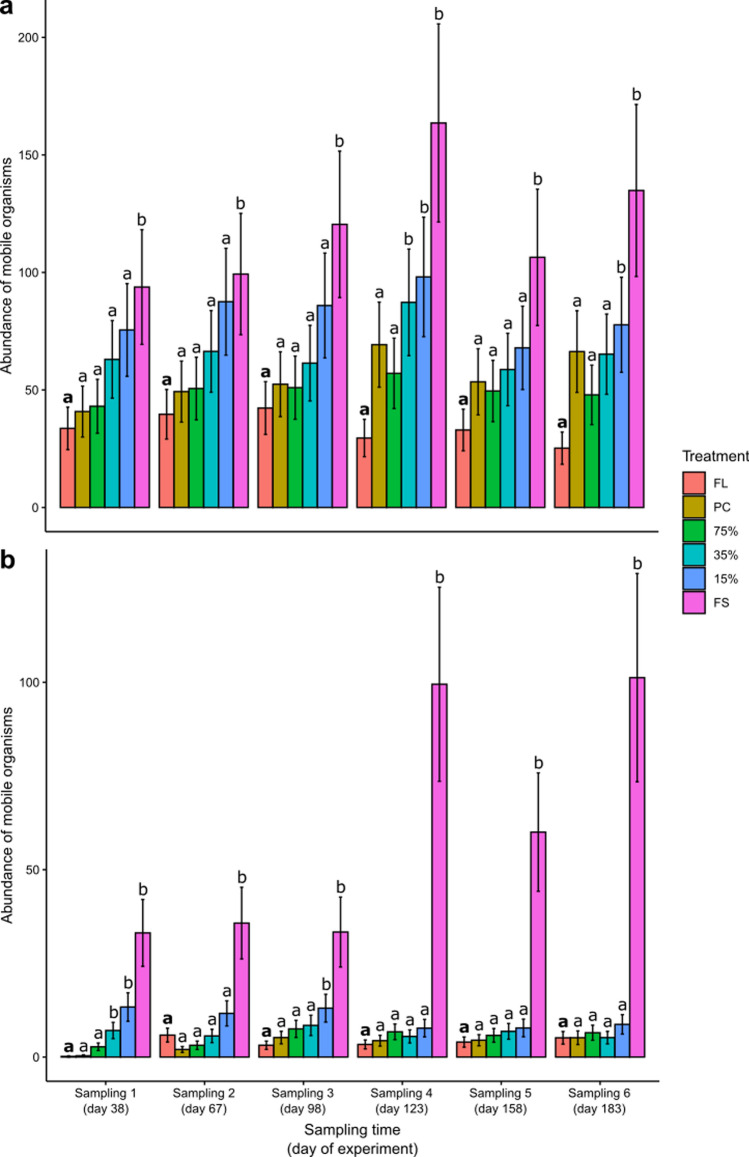
Abundances of mobile organisms for each treatment within **a** pools and on **b** emergent rock at CB North. *FL* Full light, *PC* Procedural control, 75% = 75% light transmission, 35% = 35% light transmission, 15% = 15% light transmission, and FS = full shade (*N* = 4–5 per treatment per sampling time, see Table S2). Note the different x-axes scales. Error bars are model predicted means and standard errors. Different letters indicate significant differences among the full light treatment (highlighted in bold) and other light treatments

The abundance of mobile organisms also varied interactively among treatments, habitats, and time (Table 2). Within pools (Fig. 5a) and on emergent rock (Fig. 5b), the abundance of mobile taxa was significantly higher in fully shaded plots compared to plots in full sunlight throughout the experiment (see supplementary material 2 for results for model results), with fully shaded plots having at least more than double the abundance of mobile organisms than those in full sunlight. On some sampling occasions, plots under 35% and 15% light transmission also had significantly more mobile organisms than those under full sunlight conditions, but this was not consistent across time (Fig. 5, see supplementary material 2 for results for model results).

When comparing the ratio (i.e., the effect size) of the different light treatments on the abundance of mobile organisms compared to full sunlight between the two habitats, reducing light transmission to ≤ 35 increased abundances of mobile organisms significantly more on emergent rock than in pools, but only at the first time of sampling (Fig. S5, see supplementary material 2 for results for model results). At two additional sampling times (samplings 4 + 5), the effect size of fully shading plots was again greater on emergent rock than in pools (Fig. S5, see supplementary material 2 for results for model results).

**Fig. 6:**
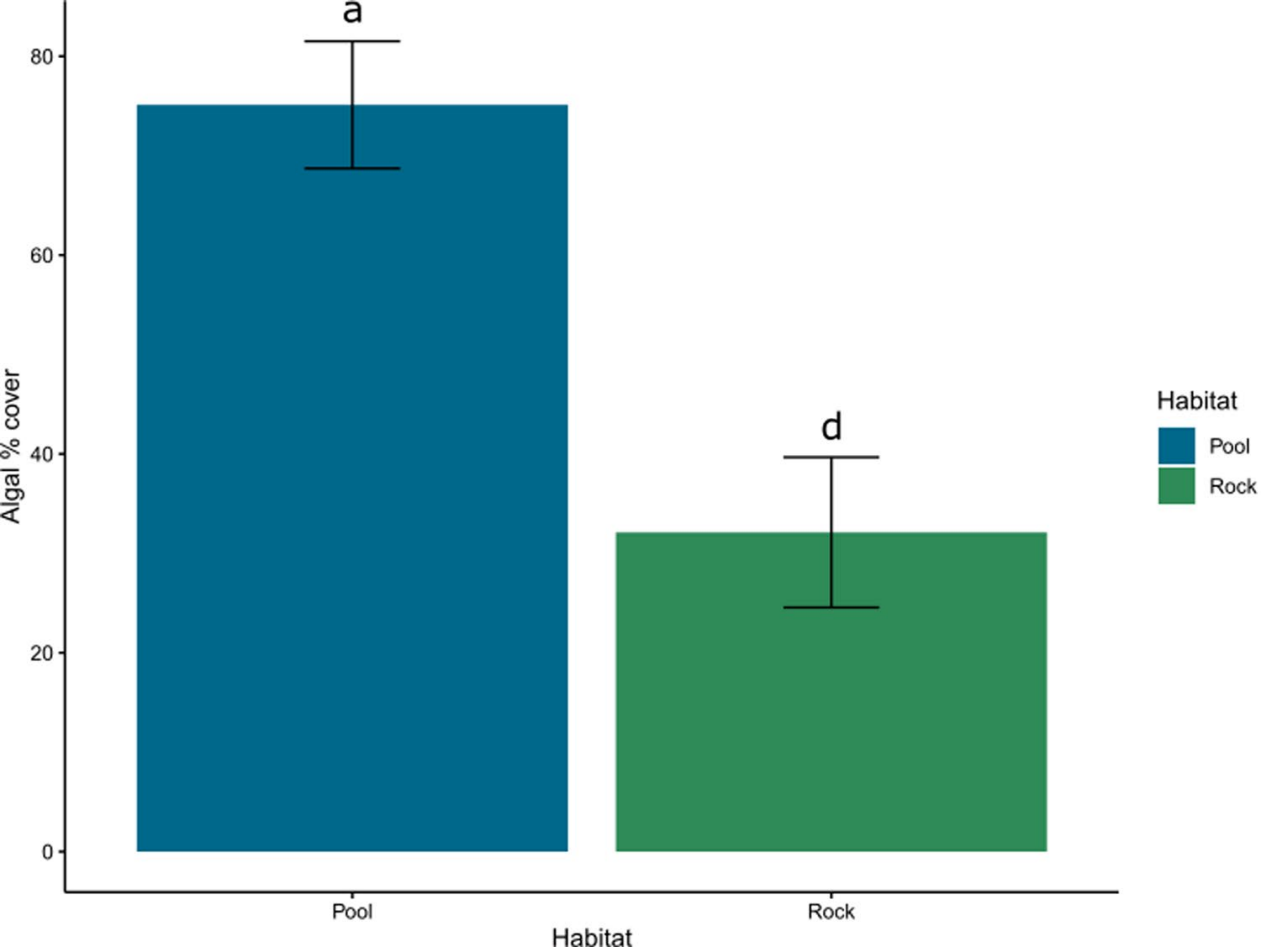
Algal % cover after 6 months for each habitat, averaged across treatments at CB North (*N* = 28–29 per habitat, see Table S2). Error bars are model predicted means and standard errors

After six months, algal cover varied between habitats, but not among treatments (Table 1), with pools supporting more than double the algal cover than emergent rock (Fig. 6, see supplementary material 2 for results for model results). There were more sessile taxa in pools than on emergent rock regardless of light treatment (Table S5); however, some sessile taxa were limited to specific light treatments. For example, the coralline algae *Corallina* spp. was only found under full light conditions and the procedural control in pools, whereas anemones and barnacles were only found in fully shaded pools (Table S5).

#### Cape Banks (CB) East

**Fig. 7:**
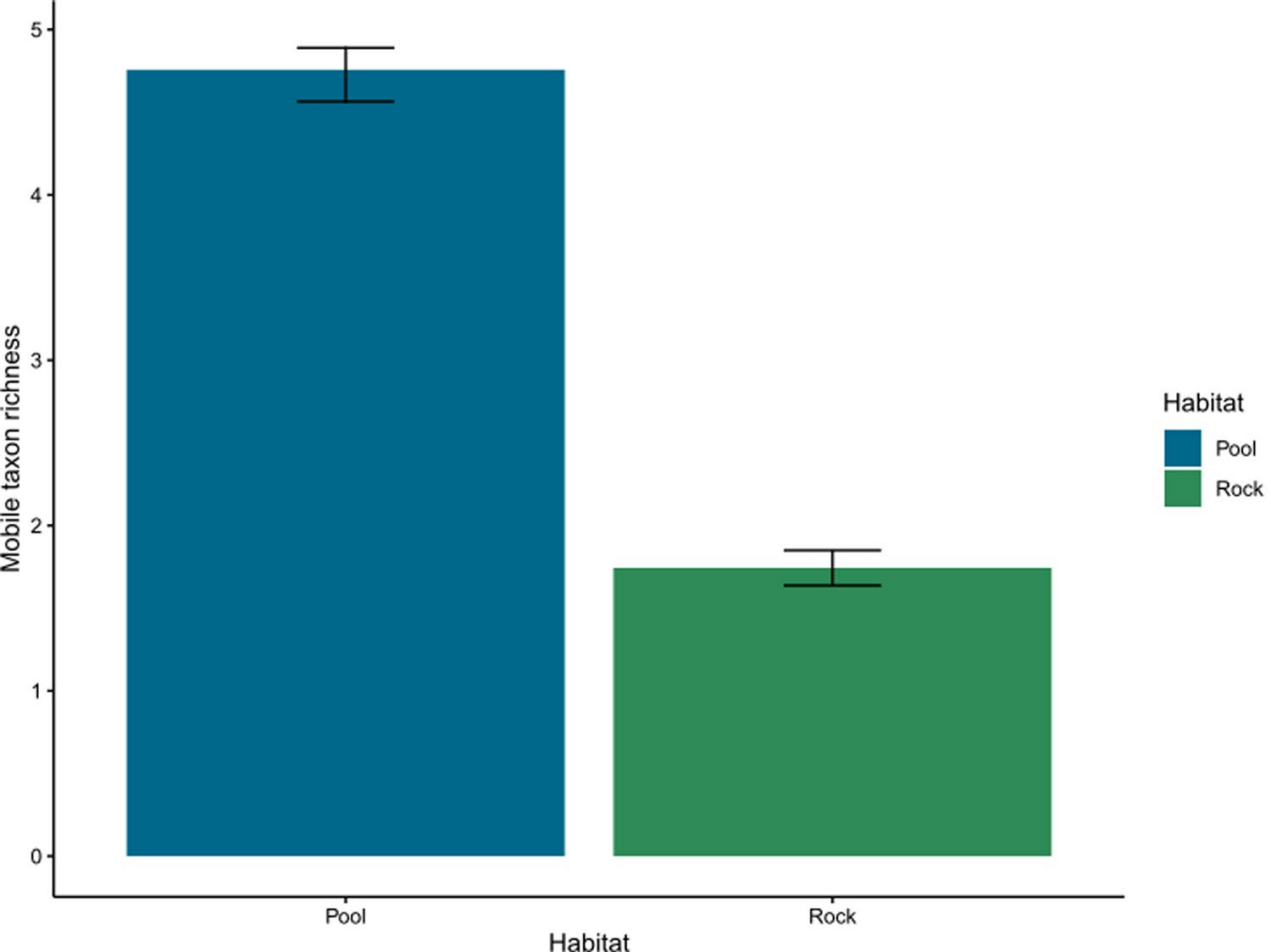
Mobile taxon richness for each habitat, averaged across treatments and time at CB East (*N* = 153–164 per habitat, see Table S2). Error bars are model predicted means and standard errors

Mobile taxon richness was significantly higher (more than double) in pools compared to emergent rock, whereas there was no effect of light treatment or sampling time (Table 2; Fig. 7, see supplementary material 2).

**Fig. 8:**
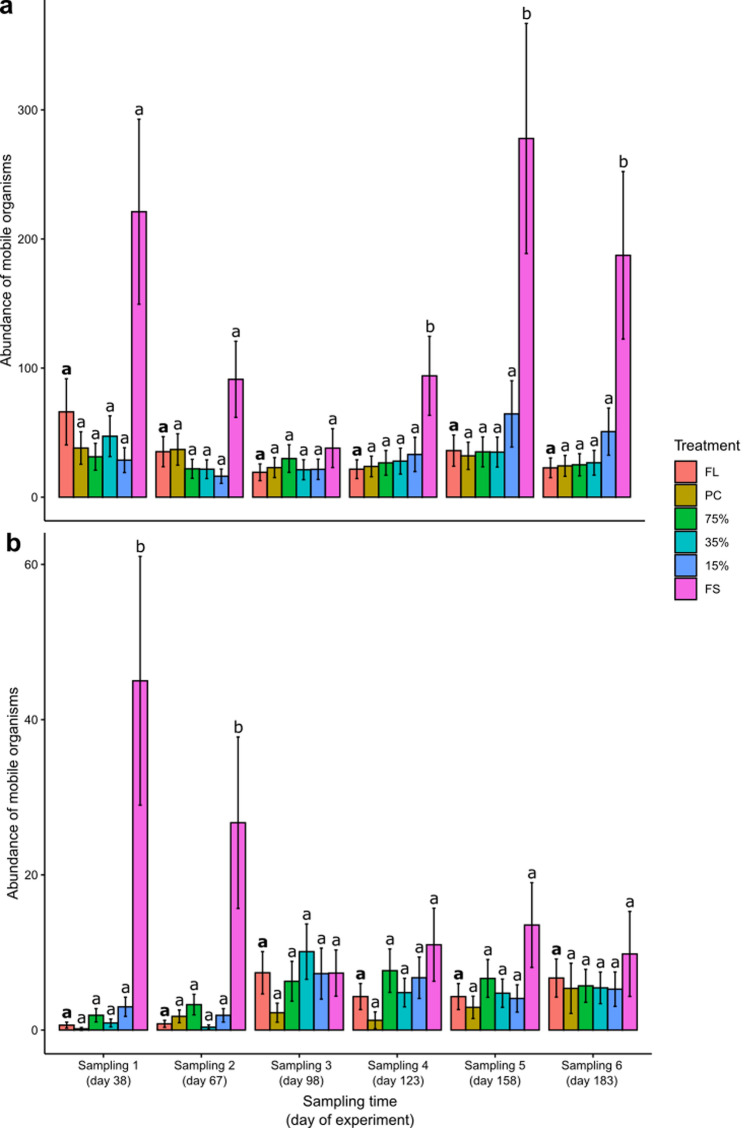
Abundances of mobile organisms for each treatment within **a** pools and on **b** emergent rock at CB East. *FL* full light, *PC* procedural control, 75% = 75% light transmission, 35% = 35% light transmission, 15% = 15% light transmission, and FS = full shade (*N* = 1–5 per treatment per sampling time, see Table S2). Note the different x-axes scales. Error bars are model predicted means and standard errors. Different letters indicate significant differences among the full light treatment (highlighted in bold) and other light treatments

The abundance of mobile organisms varied interactively among treatments, habitats, and time (Table 2). When comparing light treatments at each sampling time within pools, there was a general trend of full shade having consistently higher abundances than the full light treatment. However, this was only significant at some sampling times (Fig. 8) (see supplementary material 2 for results for model results). At those sampling times, pools in full sunlight supported 12–23% of the abundance mobile organisms than fully shaded pools (100%, mean = 94–278 mobile organisms), and plots in full sunlight on emergent rock supported 2–4% of the abundance of mobile organisms than those in full shade (100%, mean = 27–45 mobile organisms). This was mainly driven by higher abundances of the gastropod species *N. melanotragus* within pools, and *N. melanotragus, Bembicium nanum,* and *Austrocochlea* spp. on emergent rock.

We found that the effects of reducing light levels to ≤ 15% were significantly stronger on emergent rock than in pools, when looking at the ratio (i.e., the effect size) of the different light treatments on the abundance of mobile organisms compared to the full light treatment among the two habitats. This was, however, limited to the first and second sampling time, which occurred during the Austral summer when temperatures were > 30 °C on sampling days (Table S5), and were limited to the full shade treatment (Fig. S11, see supplementary material 2 for results for model results).

**Fig. 9:**
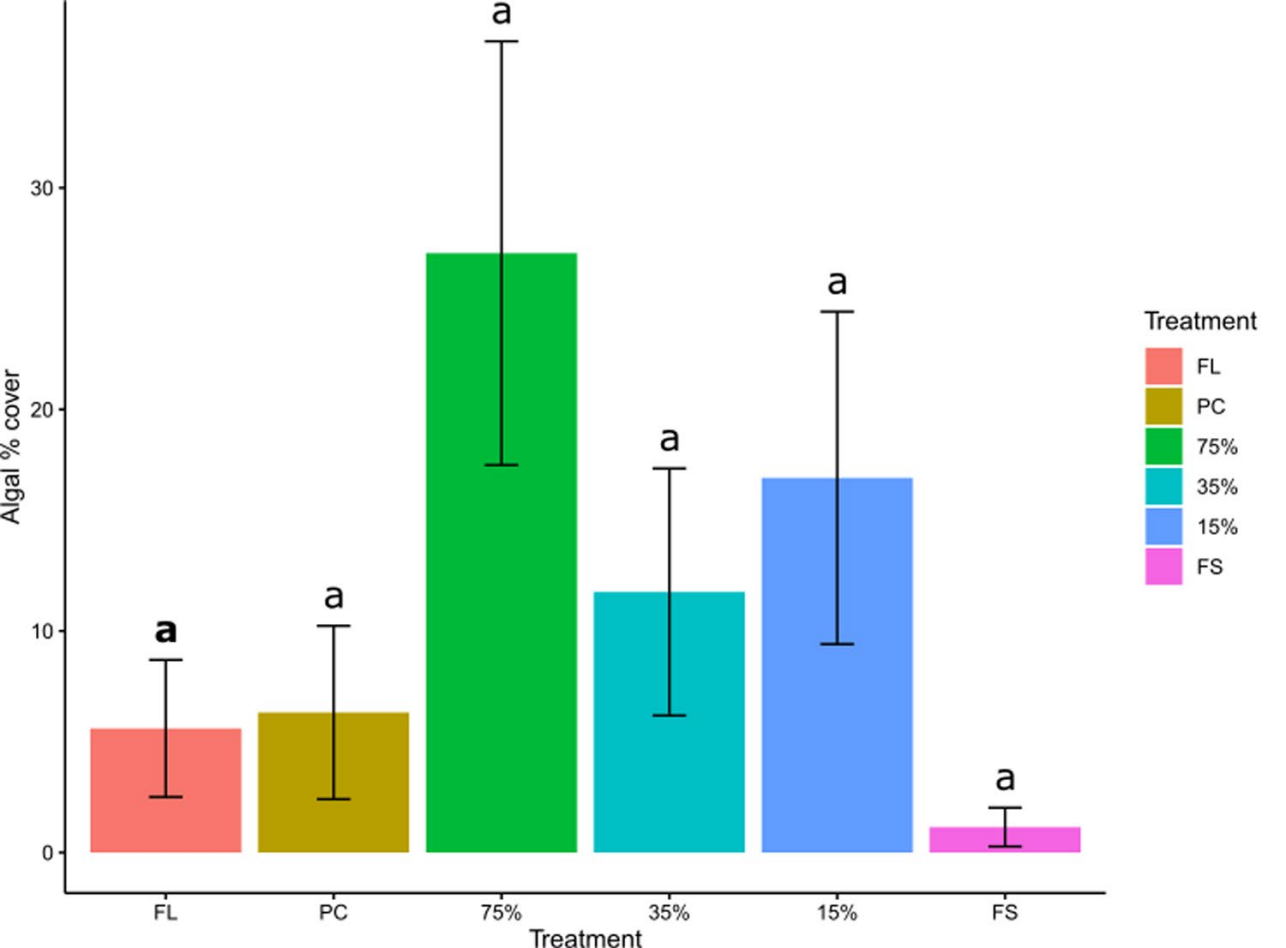
Algal % cover after 6 months for each treatment at CB East, averaged across habitats. *FL* full light, *PC* procedural control, 75% = 75% light transmission, 35% = 35% light transmission, 15% = 15% light transmission, *FS* full shade (*N* = 53–60 per treatment, see Table S2). Error bars are model predicted means and standard errors

After 6 months, the highest algal cover was observed in plots of 75% light transmission, and the least in fully shaded plots (Fig. 9). Effects of shading on algal cover varied with treatment, but not between habitats (Table 2). However, pairwise comparisons failed to detect significant differences (see supplementary material 2 for results for model results). Across all replicates, the most sessile taxa were found in procedural control (in pools) and 75% light transmission plots (in pools and on emergent rock) (Table S8).

## Discussion

This experiment tested the effects of light intensity on both sessile and mobile rocky shore communities at different types of habitats and found that effects of reducing light levels (i.e., shading) were generally stronger on emergent rock than in pools, as hypothesised. Furthermore, although we did find reduced temperature in rock pools than on exposed rocks, within these habitats, we found no differences of temperature among light intensities. Mobile richness and abundances generally increased with decreasing light levels in both habitats, in line with our hypothesis. We also found that, contrary to our prediction, algal cover was highest under intermediate light conditions, but effects were only observed at one of the studied sites. These findings suggest that effects of light and habitat types on intertidal communities are complex, and that the provision of a combination of different light levels is likely to best produce the current variability of light conditions on natural rocky shores to benefit the recruitment of both sessile and mobile communities.

Mobile taxon richness and abundances were generally greater in pools than on emergent rock, which could be due to the lower temperatures found in water than on emergent rock. However, differences in effect sizes of shade treatments compared to full light were not always significantly different between habitat types, with significant differences coinciding with samplings of high temperature (> 30 °C), where water retained in rock pools would have likely given additional relief from desiccation stress (Metaxas and Scheibling 1993). This effect though was mainly limited to comparisons in effect sizes between full sunlight and the full shade treatment. It is likely that other light treatments were mostly avoided by mobile species during periods of high environmental stress (Garrity 1984). These results corroborate other studies that found indicate that habitats that provide shade and water (i.e., rock pools) are crucial microhabitats for mobile species, increasing their richness and abundances, especially during hot weather periods in summer where mobile organisms are close to their temperature thresholds and heat and desiccation stress are highest (Bishop et al. 2022; Garrity 1984; Takada 1999).

The effect of reduced light intensity on mobile abundances was immediate and substantial in both habitats and continued throughout the experiment. This suggests that responses to light treatments were either related to the lower light levels and associated reduction in environmental stressors (such as temperature), and/or due to shelter from predators provided by the plates (which prevented for example, fish or larger crabs to access the experimental plots) rather than due to potential indirect effects related to food availability. This is because the substrate had been cleared at the start of the experiment and differences in mobile abundances continued to differ up to the last sampling time, where no differences in algal abundances among treatments were found. Higher abundances of mobile organisms under darker treatments could be a result of darker shades providing a visual cue for darker, and thus likely cooler, habitat, which, in turn, could have influenced species distributions among treatments. Additionally, mobile organisms in shaded microhabitats experience reduced heat and desiccation stress (Garrity 1984; Gray and Hodgson 2004), and similar trends have been reported in several other studies (Fairweather 1988; Moreira et al. 2007; Takada 1999). The lack of an effect of treatments of higher light intensities, in particular within the 75% light transmission treatment, can be due to lower differences in light transmissions between these treatments and full light, suggesting that there is a threshold of light intensity reduction (and likely an associated reduction in light-related stressors like heat) to which mobile organisms respond.

While no differences in effects of temperature between different light treatments were found for either habitat in this study, this may be due to how temperature measurements were taken, i.e., very punctual, short-term measurements within a ~ 3–4-h window around low tide, at different times of the day. Long-term and/or continuous measurements (i.e., throughout the day/night) might have shown different patterns and/or stronger associations between light intensity and temperature. Furthermore, newer technologies such as thermal imagery or biomimetic loggers could improve assessments of effects of shade treatments as they reflect more realistically the conditions being experienced by the organisms themselves (Helmuth and Hofmann 2001; Lathlean and Seuront 2014) and should be considered in future studies.

There was twice the cover of algae in plots with intermediate light levels compared to low or high light levels’ treatments at CB East in both habitats. These differences were, however, not significant, likely due to high variability among replicates. Optimal performance of aquatic algae under intermediate light conditions likely results from a trade-off in obtaining maximal light for photosynthesis (Ruiz and Romero 2001) while minimising light stress due to harmful UV light (Pessoa 2012; Powles 1984). At CB North, there was no difference in algal cover among light treatments, but pools supported greater algal cover (approximately double) than emergent rock plots, likely due to reduced desiccation stress in rock pools. The lack of significant differences among light treatments in both types of habitats may be a result of the encrusting algae *Ralfsia* spp. being the dominant algae in all light treatments, which can rapidly colonise bare plots (Bulleri 2005) and, as an encrusting algae, is likely adapted, to some extent, to low-light conditions (Markager and Sand-Jensen 1992).

Overall, abundances of mobile taxa and algal cover were higher at CB North, which can be linked to the different shore levels at which the treatment plots were located. Sampled plots at CB East were located higher on the shore, which increases desiccation and heat stress due to extended exposure time at upper shore levels (Huggett and Griffiths 1986; Morris and Taylor 1983). Additionally, some of the experimental plots at this site did not support any biota before the experiment, suggesting that potential mitigation of physiological stressors by shading may have not been sufficient for biota at this height on shore. Other differences, such as wave exposure, which was higher at CB East, may have also contributed to an overall greater abundance of algae and mobile abundances at this site (McQuaid 1985).

Although not statistically tested, there was little variability in sessile taxon richness across light treatments. On emergent rock, this was mainly due to an overall low number of taxa, with the brown encrusting algae *Ralfsia* spp. being the dominant alga. In contrast, rock pools had up to seven taxa across replicates in each treatment. Mobile organisms were most abundant under full light conditions, which could have led to increased grazing and thus reduced algal cover or recruitment under these treatments (Anderson and Underwood 1997; Bellgrove et al. 2014; Underwood 1980). However, since final cover and diversity of algae were similar among low light and full light in both habitats, it is unlikely that grazing limited the succession and growth of algae. Nevertheless, some sessile taxa only occurred in particular treatments and were limited to rock pools. For example, the red algae *Corallina* spp. occurred solely in rock pools of high light intensity, whereas barnacles and anemones mainly occurred in fully shaded rock pools, which suggest that some species need particular light conditions to grow. Invertebrate larvae are negatively phototactic, settling into the darker spaces where they are not outcompeted by algae (Miller and Etter 2008; Schaefer et al. 2023). Greater sessile taxon richness in rock pools was likely related to lower tolerances of desiccation by some of the species. Additionally, nutrients supplied by incoming tides are retained in rock pool water, which gives algae more time to absorb nutrients and may contribute to greater productivity of algae in rock pools than emergent rock.

Experimental plots here were approx. 0.3–1 m apart, and gastropod species found in this study are known to have daily movements within or even greater than this range (Underwood 1977). Therefore, the strong effects observed here of full shade relative to other treatments might be due to a lack of independence among plots regarding mobile taxa. If, for instance, most animals chose the ‘best’ habitat available (which in this case would be the full shaded plots), there would be fewer animals to ‘inhabit’ the habitats under the other treatments. Further testing with experimental plots further apart is necessary to fully disentangle the effects of the different light treatments on mobile species. Furthermore, the height of artificial shades may have played an important part in the response of mobile organisms, since shades were placed only a few centimetres above the rock, which may have inhibited access by some larger predators like fish and crabs, and whether responses would be similar if shades were placed further away from the rock bed remains to be tested.

In many natural environments, visual predation is strongly related to ambient light, with animals feeding either during the day, night or dusk/dawn, depending on their physiology and/or adaptation regarding lightscapes (James and Heck 1994). Similarly, habitat complexity has been shown to influence the distribution and abundance of organisms, affecting several ecological processes (MacArthur and MacArthur 1961 and others). Critically, habitat complexity can influence light intensities at different spatial scales—e.g., vegetation on land and underwater can reduce the amount and intensity of light reaching the substrate underneath their canopies at both small (i.e., individual plants) to large scales (forests) (Parker et al. 1995; Reed and Foster 1984). For example, increasing structural complexity decrease visual contact and mobility of fish predators, as vegetation acts as a deterrent to predator movement (Einfalt et al. 2012; James and Heck 1994; Sass et al. 2006; Savino and Stein 1989). Here, we found that changes in light intensity had stronger effects on species richness on less complex habitats (e.g., emergent rock) than on more complex ones, such as rock pools. With increasing degradation and homogenization of habitats as well as changes in natural light cycles (due to the use of artificial illumination), these results not only provide important insights of the mechanisms driving changes on modified habitats but have important implications for management and conservation of habitats. Our findings suggest, for example, that in urbanised coastal areas, while the addition of small, shaded microhabitats like overhangs or ledges to new or existing marine infrastructure should be considered to increase the abundance and richness of mobile organisms on these structures, large, shaded areas should be avoided, as they do not reflect natural light conditions along rocky shores and reduce algal growth. Furthermore, to maximise diversity at larger spatial scales (e.g., the entire rocky shore), varying light levels are needed. Here, we found that some taxa, such as the red algae Corallina spp., or barnacles and anemones, were only observed under particular light conditions.

## Conclusion

This study was the first one to assess the interactive effects of complexity and light, and showed that on coastal intertidal habitats, light and complexity affect assemblages both independently and interactively, and that effects varied between different taxonomic groups. Light was more important in driving mobile species richness in less complex habitats, whereas the abundance of mobile organisms was driven by different light intensities regardless of the habitat, though effects appeared to be slightly greater in simpler habitats. Algae were more diverse in submerged habitats, whereas invertebrates were more abundant in low-light environments.

This indicates that shade is an important driver of mobile species richness in simple habitats, likely through provision of refuge for species less tolerant to strong irradiances when no other protection (e.g., submersion in water) exists. The fact that effects of light on mobile abundances were similar for both habitats despite effects on species richness indicates that patterns are likely driven by a small number of species that can thrive as long as some form of protection (i.e., shade or submersion) exists. In contrast, light was less important for taxa that are more prone to desiccation, such as erect algae forms.

Our results reinforce that effects of complexity and light on biotic communities are complex, and effects will vary with the taxonomic groups present, their environmental tolerances, and competitive interactions. In many ecosystems affected by human disturbance, impacts on complexity are likely to influence the light environment, and vice versa, and are likely to affect different associated communities in different ways. For example, deforestation of canopy-forming trees will reduce complexity and increase light levels for sub-canopy species. Similarly, culverting of streams reduces habitat complexity and permanently shades the water. Identifying how different taxonomic groups, such as mobile species (e.g., fish and crustaceans in riverine systems, insects, rodents, and birds in terrestrial systems) and different types of sessile communities (algae and invertebrates in freshwater systems, canopy-formers, understorey shrubs, and ground cover in terrestrial systems) are affected by complexity and light is important to not only predict impacts, but also optimise management strategies to minimise ecological impacts.

### Supplementary Information

Below is the link to the electronic supplementary material.

Supplementary file1 (DOCX 1948 KB)

Supplementary file2 (XLSX 36 KB)

Supplementary file3 (XLSX 1948 KB)

## Data Availability

The datasets used and/or analysed during the current study are available from the corresponding author on reasonable request.
