# Supplementary material for: Investigating the interactive effects of habitat type and light intensity on rocky shores
Source: Oecologia. 2024 Jul 24;205(3-4):627–42. doi: 10.1007/s00442-024-05591-2 (PMC11358318; doi:10.1007/s00442-024-05591-2)
Supplement: Supplementary file 1 — Supplementary file1 (DOCX 1948 KB) [file 442_2024_5591_MOESM1_ESM.docx]

**Investigating the interactive effects of habitat type and light intensity on rocky shores**

Nina Schaefer ^a,b*^, Katherine A. Dafforn ^a,b^, Emma L. Johnston ^a,c^, Graeme F. Clark ^a,c^, Mariana Mayer-Pinto ^a^

^a^ Centre for Marine Science and Innovation; Evolution and Ecology Research Centre, School of Biological, Earth and Environmental Sciences, University of New South Wales, Sydney, NSW 2052, Australia

^b^ School of Natural Sciences, Macquarie University, North Ryde, NSW 2109, Australia

^c^ School of Life and Environmental Sciences, University of Sydney, Camperdown, NSW 2006, Australia

* Corresponding author: Nina Schaefer ([n.schaefer@unsw.edu.au](mailto:n.schaefer@unsw.edu.au), nina.schaefer@mq.edu.au)

^[[1]](#footnote-1)^

## Supplementary materials

**Table S1** Estimated light transmission and experimental set up for each treatment (estimates are based on data from manufacturers). Abbreviations for treatments are shown in brackets

| Treatment | Light transmission | Experimental set up |
| --- | --- | --- |
| Full light (FL) | 100%, + UV | no plate |
| Procedural control (PC) | ~92%, no UV | clear plate |
| 75% | ~75%, no UV | clear plate + "Bolle Clear" film |
| 35% | ~35%, no UV | clear plate + "Octane" film |
| 15% | ~15%, no UV | clear plate + "ATP18GH" film |
| Full shade (FS) | ~0%, no UV | black plate |

**Table S2** Number of replicates per sampling for each treatment at each site. FL = Full light, PC = Procedural control, 75% = 75% light transmission, 35% = 35% light transmission, 15% = 15% light transmission, FS = Full shade

| **CB North** |  | Mobile taxa | | | | | |  | Sessile taxa | | | | | |
| --- | --- | --- | --- | --- | --- | --- | --- | --- | --- | --- | --- | --- | --- | --- |
| **Rock pool** |  | FL | PC | 75% | 35% | 15% | FS |  | FL | PC | 75% | 35% | 15% | FS |
| Sampling 1 (day 38) |  | 5 | 5 | 5 | 5 | 5 | 5 |  | 5 | 5 | 5 | 5 | 5 | 5 |
| Sampling 2 (day 67) |  | 5 | 5 | 5 | 5 | 5 | 5 |  | 5 | 5 | 5 | 5 | 5 | 5 |
| Sampling 3 (day 98) |  | 5 | 5 | 5 | 5 | 5 | 5 |  | 5 | 5 | 5 | 5 | 5 | 5 |
| Sampling 4 (day 123) |  | 5 | 5 | 5 | 5 | 5 | 5 |  | 5 | 5 | 5 | 5 | 5 | 5 |
| Sampling 5 (day 158) |  | 5 | 5 | 5 | 5 | 5 | 4 |  | 5 | 5 | 5 | 5 | 5 | 5 |
| Sampling 6 (day 183) |  | 5 | 5 | 5 | 5 | 5 | 4 |  | 5 | 5 | 5 | 5 | 5 | 4 |
|  |  |  |  |  |  |  |  |  |  |  |  |  |  |  |
| **CB East** |  | Mobile taxa | | | | | |  | Sessile taxa | | | | | |
| **Rock pool** |  | FL | PC | 75% | 35% | 15% | FS |  | FL | PC | 75% | 35% | 15% | FS |
| Sampling 1 (day 38) |  | 3 | 5 | 5 | 5 | 5 | 5 |  | 3 | 5 | 5 | 5 | 5 | 5 |
| Sampling 2 (day 67) |  | 5 | 5 | 5 | 5 | 5 | 5 |  | 5 | 5 | 5 | 5 | 5 | 5 |
| Sampling 3 (day 98) |  | 5 | 5 | 4 | 4 | 4 | 3 |  | 5 | 5 | 5 | 4 | 5 | 4 |
| Sampling 4 (day 123) |  | 5 | 5 | 4 | 4 | 3 | 5 |  | 5 | 5 | 5 | 4 | 3 | 5 |
| Sampling 5 (day 158) |  | 5 | 5 | 5 | 5 | 3 | 5 |  | 5 | 5 | 5 | 5 | 4 | 5 |
| Sampling 6 (day 183) |  | 5 | 5 | 5 | 4 | 4 | 4 |  | 5 | 5 | 5 | 4 | 4 | 4 |
|  |  |  |  |  |  |  |  |  |  |  |  |  |  |  |
|  |  |  |  |  |  |  |  |  |  |  |  |  |  |  |
|  |  |  |  |  |  |  |  |  |  |  |  |  |  |  |
| **CB North** |  | Mobile taxa | | | | | |  | Sessile taxa | | | | | |
| **Emergent rock** |  | FL | PC | 75% | 35% | 15% | FS |  | FL | PC | 75% | 35% | 15% | FS |
| Sampling 1 (day 38) |  | 5 | 5 | 5 | 5 | 5 | 5 |  | 5 | 5 | 5 | 5 | 5 | 5 |
| Sampling 2 (day 67) |  | 5 | 5 | 5 | 5 | 5 | 5 |  | 5 | 5 | 5 | 5 | 5 | 5 |
| Sampling 3 (day 98) |  | 5 | 5 | 5 | 4 | 5 | 4 |  | 5 | 5 | 5 | 5 | 5 | 4 |
| Sampling 4 (day 123) |  | 5 | 5 | 5 | 5 | 5 | 5 |  | 5 | 5 | 5 | 5 | 5 | 5 |
| Sampling 5 (day 158) |  | 5 | 5 | 5 | 5 | 5 | 5 |  | 5 | 5 | 5 | 5 | 5 | 5 |
| Sampling 6 (day 183) |  | 4 | 5 | 5 | 5 | 5 | 4 |  | 5 | 5 | 5 | 5 | 5 | 4 |
|  |  |  |  |  |  |  |  |  |  |  |  |  |  |  |
| **CB East** |  | Mobile taxa | | | | | |  | Sessile taxa | | | | | |
| **Emergent rock** |  | FL | PC | 75% | 35% | 15% | FS |  | FL | PC | 75% | 35% | 15% | FS |
| Sampling 1 (day 38) |  | 5 | 5 | 5 | 5 | 5 | 5 |  | 5 | 5 | 5 | 5 | 5 | 5 |
| Sampling 2 (day 67) |  | 5 | 5 | 5 | 5 | 5 | 3 |  | 5 | 5 | 5 | 5 | 5 | 4 |
| Sampling 3 (day 98) |  | 5 | 3 | 4 | 5 | 3 | 4 |  | 5 | 5 | 5 | 5 | 4 | 5 |
| Sampling 4 (day 123) |  | 5 | 2 | 5 | 5 | 4 | 3 |  | 5 | 4 | 5 | 5 | 4 | 5 |
| Sampling 5 (day 158) |  | 5 | 4 | 5 | 5 | 4 | 4 |  | 5 | 5 | 5 | 5 | 5 | 5 |
| Sampling 6 (day 183) |  | 5 | 2 | 5 | 5 | 1 | 2 |  | 5 | 3 | 5 | 5 | 4 | 3 |

**
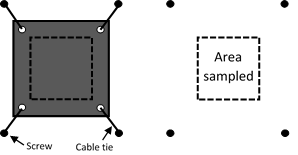
**

**Fig. S1** Shaded area (20 x 20 cm) versus sampled area (~ 15 x 15 cm) of experimental plots on emergent rock

**Table S2** Analysis of deviance table for light measurements for the interactive and additive model

| **Analysis of Deviance Table (Type III tests)** | Chisq | Df | Pr(>Chisq) |
| --- | --- | --- | --- |
| (Intercept) | 4182.46 | 1 | **<2.2E-16** |
| Time | 121.00 | 4 | **<2.2E-16** |
| Treatment | 744.46 | 5 | **<2.2E-16** |
| Habitat | 8.31 | 1 | **0.0039** |
| Treatment*Habitat | 7.62 | 5 | 0.1784 |
| **Analysis of Deviance Table (Type II tests)** |  |  |  |
| Time | 121.52 | 4 | **<2.2E-16** |
| Treatment | 1696.952 | 5 | **<2.2E-16** |
| Habitat | 27.735 | 1 | **1.391E-07** |
|  |  |  |  |

**Table S3** Contrasts among light treatments for light measurements on the response-scale. FL = Full light, PC = Procedural control, 75% = 75% light transmission, 35% = 35% light transmission, 15% = 15% light transmission, FS = Full shade. Results are averaged across time and habitat

| **Pairwise comparisons** | ratio | SE | df. | null | t.ratio | p.value |
| --- | --- | --- | --- | --- | --- | --- |
| FL - PC | 1.14 | 0.114 | 112 | 1 | 1.356 | 0.7527 |
| FL – 75% | 1.21 | 0.120 | 112 | 1 | 1.894 | 0.44141 |
| FL – 35% | 1.81 | 0.179 | 112 | 1 | 6.004 | **<0.0001** |
| FL – 15% | 2.96 | 0.293 | 112 | 1 | 10.971 | **<0.0001** |
| FL - FS | 32.54 | 3.271 | 112 | 1 | 34.644 | **<0.0001** |
| PC – 75% | 1.05 | 0.105 | 112 | 1 | 0.527 | 0.9950 |
| PC – 35% | 1.58 | 0.157 | 112 | 1 | 4.614 | **0.0002** |
| PC – 15% | 2.58 | 0.257 | 112 | 1 | 9.552 | **<0.0001** |
| PC - FS | 28.42 | 2.870 | 112 | 1 | 33.153 | **<0.0001** |
| 75% – 35% | 1.50 | 0.148 | 112 | 1 | 4.105 | **0.0011** |
| 75% – 15% | 2.45 | 0.243 | 112 | 1 | 9.072 | **<0.0001** |
| 75% - FS | 26.97 | 2.711 | 112 | 1 | 32.776 | **<0.0001** |
| 35% – 15% | 1.63 | 0.161 | 112 | 1 | 4.980 | **0.0001** |
| 35% - FS | 17.97 | 1.802 | 112 | 1 | 28.811 | **<0.0001** |
| 15% - FS | 11.00 | 1.103 | 112 | 1 | 23.912 | **<0.0001** |


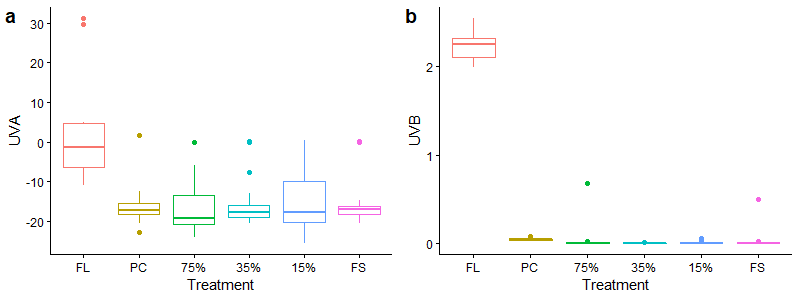


**Fig. S2** (a) UVA and (b) UVB measurements under full light and under experimental shades. FL = Full light, PC = Procedural control, 75% = 75% light transmission, 35% = 35% light transmission, 15% = 15% light transmission, FS = Full shade

**Table S4** Analysis of deviance table for temperature measurements for the interactive and additive model

| **Analysis of Deviance Table (Type III tests)** | Chisq | Df | Pr(>Chisq) |
| --- | --- | --- | --- |
| (Intercept) | 7379.42 | 1 | **<2.2E-16** |
| Time | 3006.35 | 4 | **<2.2E-16** |
| Treatment | 3.35 | 5 | 0.6468 |
| Habitat | 2.27 | 1 | 0.1319 |
| Treatment*Habitat | 4.85 | 5 | 0.4347 |
| **Analysis of Deviance Table (Type II tests)** |  |  |  |
| Time | 3009.01 | 4 | **<2.2E-16** |
| Treatment | 6.38 | 5 | 0.27119 |
| Habitat | 4.02 | 1 | **0.04495** |
|  |  |  |  |

**Fig. S3** Temperature (^o^ Celsius) under full light and under experimental shades in rock pools and on emergent rock at each sampling time. FL = Full light, PC = Procedural control, 75% = 75% light transmission, 35% = 35% light transmission, 15% = 15% light transmission, FS = Full shade (N=5 per treatment)


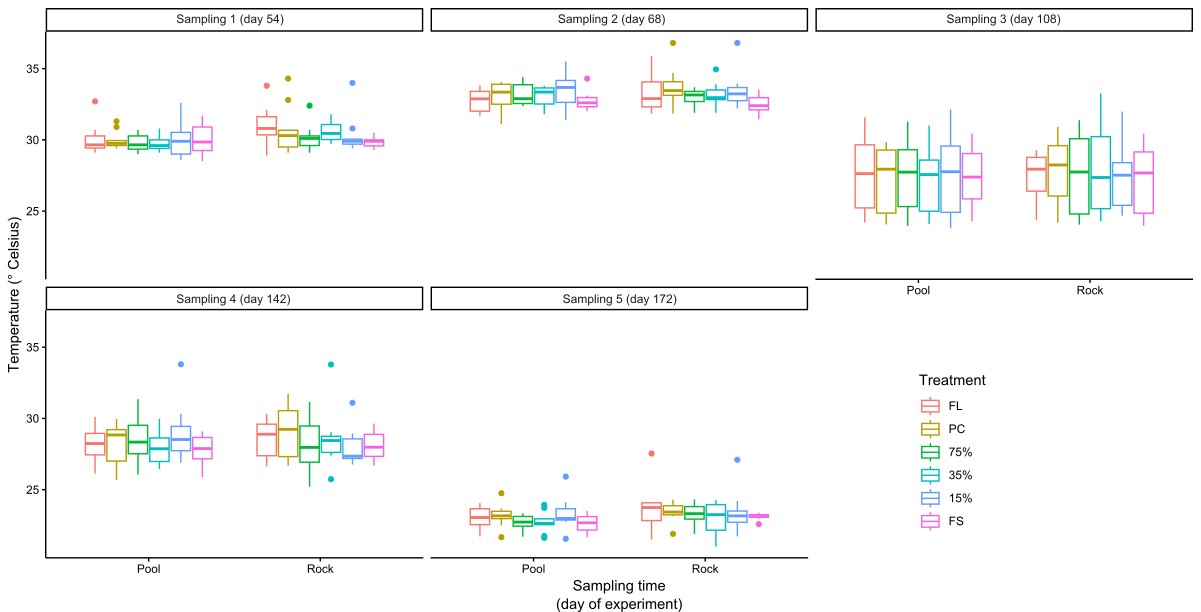


**Table S5** Temperature (minimum and maximum, ^o^Celsius) at Sydney Airport during the experiment. Data highlighted in bold are start and sampling dates. Data accessed and exported from the Bureau of Meteorology on 25.03.2023

|  | Dec-16 | | Jan-17 | | Feb-17 | | Mar-17 | | Apr-17 | | May-17 | | Jun-17 | |
| --- | --- | --- | --- | --- | --- | --- | --- | --- | --- | --- | --- | --- | --- | --- |
|  | Min | Max | Min | Max | Min | Max | Min | Max | Min | Max | Min | Max | Min | Max |
| 1 | 18.7 | 30.9 | 21.6 | 27.2 | 19.9 | 23.8 | 19.8 | 24.9 | 14.9 | 24.8 | 13.4 | 26.4 | 7.9 | 16.1 |
| 2 | 19.3 | 35.7 | 20.9 | 24.9 | 19.9 | 25.2 | 20 | 26.2 | 18 | 21 | 14.3 | 24.6 | 8.2 | 16.6 |
| 3 | 20.3 | 25.9 | 18.6 | 24.1 | 21.9 | 28.4 | 20.2 | 25.1 | 14.6 | 21.9 | 15.6 | 18.6 | 11 | 17 |
| 4 | **19.2** | **28.1** | 19.4 | 26 | 22.2 | 32.3 | 19.6 | 24.2 | 16 | 22.5 | 12 | 20.1 | 10.4 | 17.8 |
| 5 | 21.8 | 32.4 | 19.6 | 25.5 | 24 | 38.6 | 18.7 | 23.1 | 15.4 | 23.1 | 13.2 | 23.3 | **9.1** | **20.4** |
| 6 | 20 | 24.8 | 19.9 | 27.1 | 24.3 | 28.9 | 19.4 | 23.6 | **13.4** | **23.5** | 12.5 | 26.1 | 8.2 | 17.7 |
| 7 | 18.9 | 24.9 | 18.3 | 27.9 | 21.9 | 25.7 | 17.7 | 22.2 | 14.3 | 24.2 | 14.5 | 21 | 8.6 | 17.5 |
| 8 | 20.6 | 29.6 | 19.2 | 29.9 | 20.1 | 28 | 17.3 | 23.9 | 14.8 | 26 | 10.5 | 17.9 | 11.8 | 17.6 |
| 9 | 18.4 | 26.8 | 21 | 29.9 | **21.6** | **32.2** | 18 | 23.9 | 15.6 | 29.4 | 10.6 | 18.8 | 10.9 | 18.6 |
| 10 | 16.2 | 24 | 21.4 | 35.3 | 23.1 | 42.9 | 17.8 | 23.5 | 12.2 | 19.4 | 10.5 | 19.5 | 13.7 | 17.9 |
| 11 | 19.6 | 27.1 | **23** | **38.8** | 23 | 39 | 17 | 24.7 | 12.8 | 21.9 | **10.7** | **19.7** | 11.5 | 17.6 |
| 12 | 20.4 | 29 | 21.5 | 29 | 23.3 | 28.4 | **17.4** | **28.8** | 15.4 | 20.7 | 12.2 | 19.6 | 10.7 | 20 |
| 13 | 21.1 | 39.2 | 22.8 | 32.8 | 16.9 | 25.9 | 21.2 | 28.4 | 15.5 | 21.2 | 14.4 | 22.7 | 9.7 | 17.5 |
| 14 | 27.5 | 37.5 | 24.9 | 30 | 20.1 | 22.8 | 22.8 | 27.1 | 13.4 | 23 | 13.9 | 19.9 | 12.1 | 18.4 |
| 15 | 16.5 | 18.9 | 21.5 | 27.3 | 17.7 | 27.7 | 19.9 | 27.1 | 13.2 | 22.9 | 13 | 19.1 | 10.7 | 21.8 |
| 16 | 16.5 | 26 | 22 | 30 | 20.3 | 33.1 | 21.8 | 27.3 | 14.7 | 26.7 | 10.8 | 20.2 | 12.7 | 18.1 |
| 17 | 17.7 | 34.5 | 21 | 37.7 | 22.8 | 32.6 | 20 | 23.2 | 13.9 | 23.7 | 9.8 | 21.5 | 13.1 | 17.8 |
| 18 | 17.1 | 21.9 | 26.1 | 37.7 | 22 | 28.4 | 18.9 | 25.2 | 16.5 | 24.5 | 12.6 | 21.8 | 13.1 | 17.2 |
| 19 | 16.8 | 24.9 | 19.8 | 25 | 18.2 | 26 |  | 27.6 | 16 | 23.4 | 16.6 | 21.8 | 11.7 | 17.4 |
| 20 | 17.5 | 29.6 | 19.1 | 31.4 | 17 | 29.3 | 21.2 | 29.2 | 16.3 | 25.1 | 17.1 | 23.6 | 11.7 | 18.4 |
| 21 | 18.2 | 25.4 | 20.8 | 25.3 | 17.2 | 27 | 23.6 | 29.2 | 14.9 | 25.7 | 13.4 | 21 | 8.6 | 18.6 |
| 22 | 19.6 | 25 | 19.7 | 27.2 | 19.5 | 30.1 | 21.9 | 31.9 | 18.2 | 25.9 | 12.9 | 21 | 9.5 | 17.8 |
| 23 | 20.8 | 26.4 | 21.3 | 35.2 | 21.8 | 30.1 | 20.8 | 22.6 | 15.8 | 25.4 | 15 | 24.3 | 8.7 | 19.4 |
| 24 | 19.2 | 28.1 | 24.6 | 38.5 | 20.8 | 26 | 18.5 | 23.2 | 15.7 | 25.2 | 14.2 | 23.7 | 9.4 | 19.9 |
| 25 | 18.5 | 29.4 | 20.4 | 24.1 | 18.6 | 22.1 | 19.1 | 26.5 | 18.3 | 28.4 | 12.1 | 20.7 | 6.8 | 19.5 |
| 26 | 21.3 | 30.9 | 20.3 | 23.5 | 18.1 | 23.8 | 19.4 | 27.8 | 16 | 25.5 | 9.8 | 19 | 7.2 | 18.3 |
| 27 | 21.3 | 26.8 | 20.1 | 26.6 | 18.2 | 26.6 | 20.2 | 28.7 | 11.5 | 17.9 | 11.7 | 20.9 | 7.8 | 17.5 |
| 28 | 21.2 | 30.5 | 20.7 | 31.9 | 19.8 | 27.7 | 20.9 | 24.3 | 10.3 | 19.2 | 10.3 | 24.6 | 11 | 16.5 |
| 29 | 22.4 | 38.2 | 21.6 | 28.7 |  |  | 20 | 30.7 | 10.3 | 21 | 10.8 | 18.2 | 9.7 | 18.9 |
| 30 | 23.2 | 35.6 | 22.5 | 36.4 |  |  | 23.7 | 28.5 | 14.1 | 20.8 | 6.8 | 19 | 8.9 | 15 |
| 31 | 22.6 | 26.3 | 23.5 | 39.6 |  |  | 16.2 | 20.8 |  |  | 10.4 | 15.2 |  |  |
|  | Start | | Sampling 1 | | Sampling 2 | | Sampling 3 | | Sampling 4 | | Sampling 5 | | Sampling 6 | |

**Table S6** List of all the mobile and sessile taxa found

| **Mobile taxa** | **Sessile taxa** |
| --- | --- |
| Bembicium nanum | Ralfsia |
| Austrocochlea | Ulva |
| Nerita melanotragus | Corallina |
| Tenguella marginalba | Petalonia |
| Austrolittorina unifasciata | Colpomenia |
| Cellana tramoserica | Turfing algae |
| Montfortula rugosa | Encrusting algae 1 |
| Siphonaria | Algae 1 |
| Patelloida | Hard encapsulated egg |
| Gastropod 1 | Anemone |
| Gastropod 2 | Sponge |
| Gastropod 3 | Soft egg mass |
| Gastropod 4 | Dead barnacle |
| Gastropod 5 | Live barnacle |
| Gastropod 6 | Spirorbid polychaete |
| Gastropod 7 |  |
| Dicathais orbita |  |
| Nudibranchia |  |
| Elysia |  |
| Patiriella exigua |  |
| Brittlestar |  |
| Sea urchin |  |
| Sipharochiton pelliserpentes |  |
| Acantochiton |  |
| Ischnochiton |  |
| Crab |  |
| Flatworm |  |
| Fish |  |
| Onchidium damelii |  |


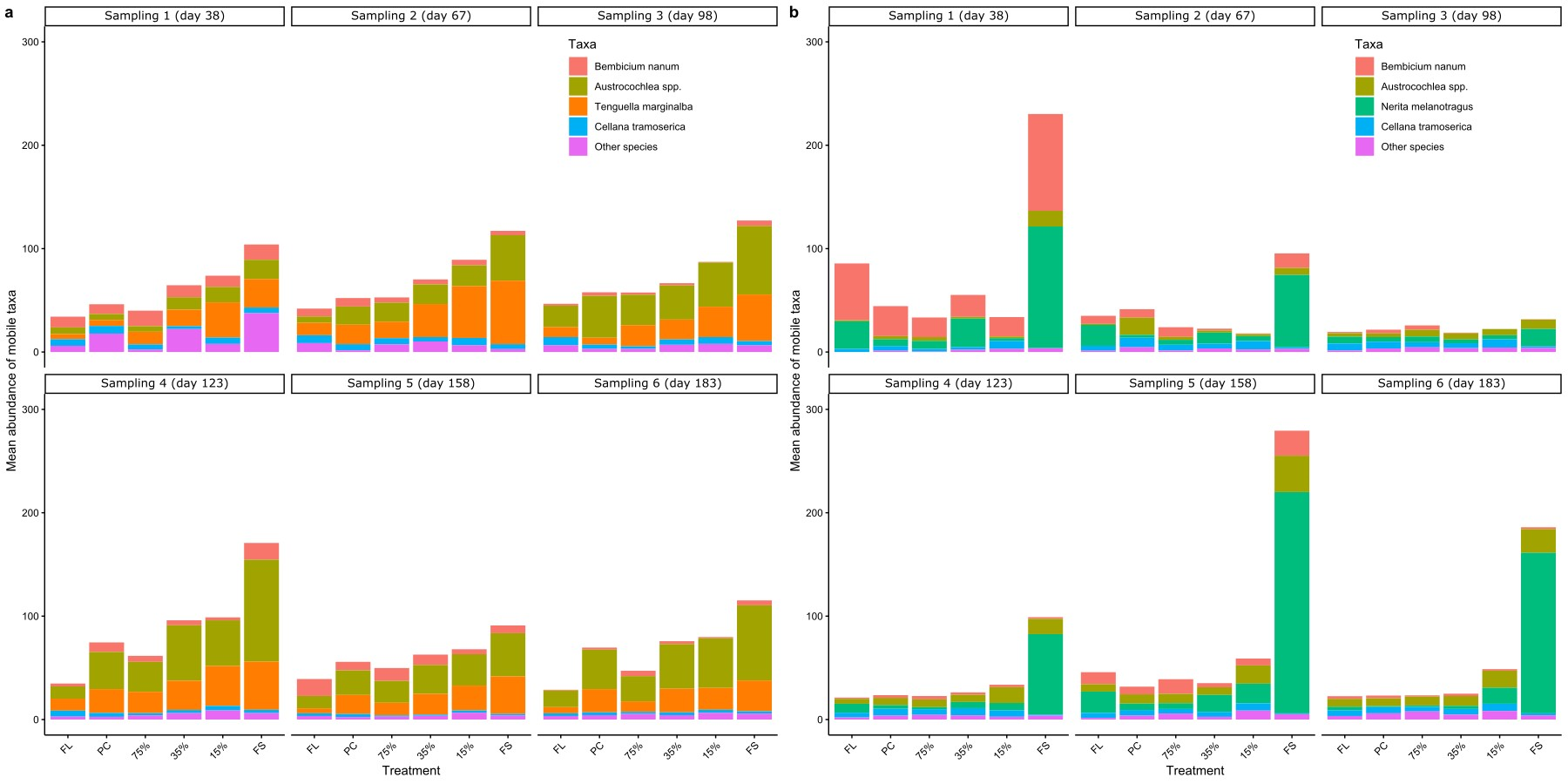


**Fig. S4** Mean abundances of the four most abundant and other taxa per sampling time at (a) Cape Banks North and at (b) Cape Banks East averaged across replicate pools. FL = Full light, PC = Procedural control, 75% = 75% light transmission, 35% = 35% light transmission, 15% = 15% light transmission, FS = Full shade (see Table S2 for number of replicates per treatment)


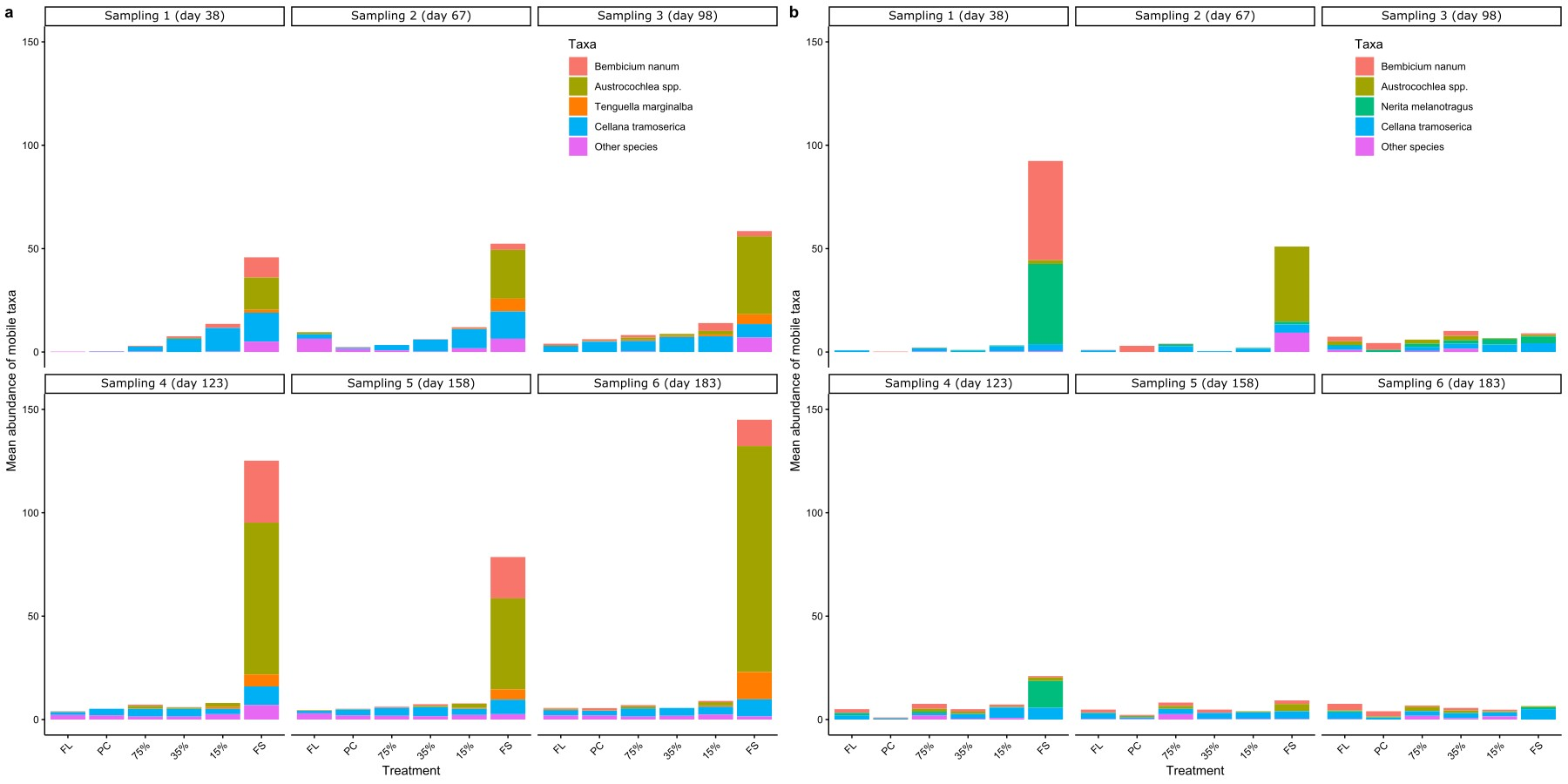
 **Fig. S5** Mean abundances of the four most abundant and other taxa per sampling time at (a) Cape Banks North and at (b) Cape Banks East averaged across replicate emergent rock. FL = Full light, PC = Procedural control, 75% = 75% light transmission, 35% = 35% light transmission, 15% = 15% light transmission, FS = Full shade (see Table S2 for number of replicates per treatment)


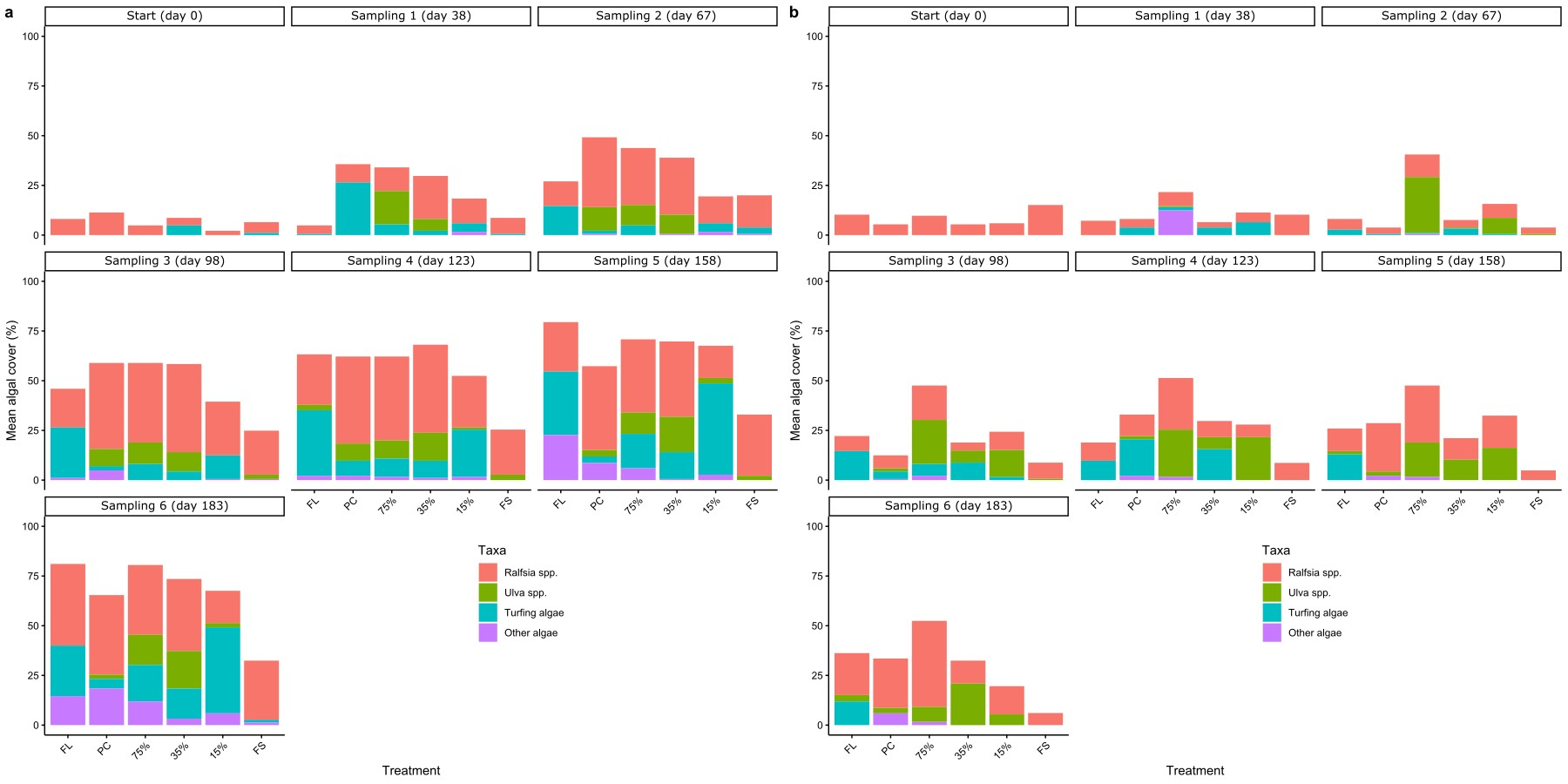
**Fig. S6** Mean algal cover (%) of the three most abundant and other taxa per sampling time at (a) Cape Banks North and at (b) Cape Banks East averaged across replicate pools. FL = Full light, PC = Procedural control, 75% = 75% light transmission, 35% = 35% light transmission, 15% = 15% light transmission, FS = Full shade (see Table S2 for number of replicates per treatment)


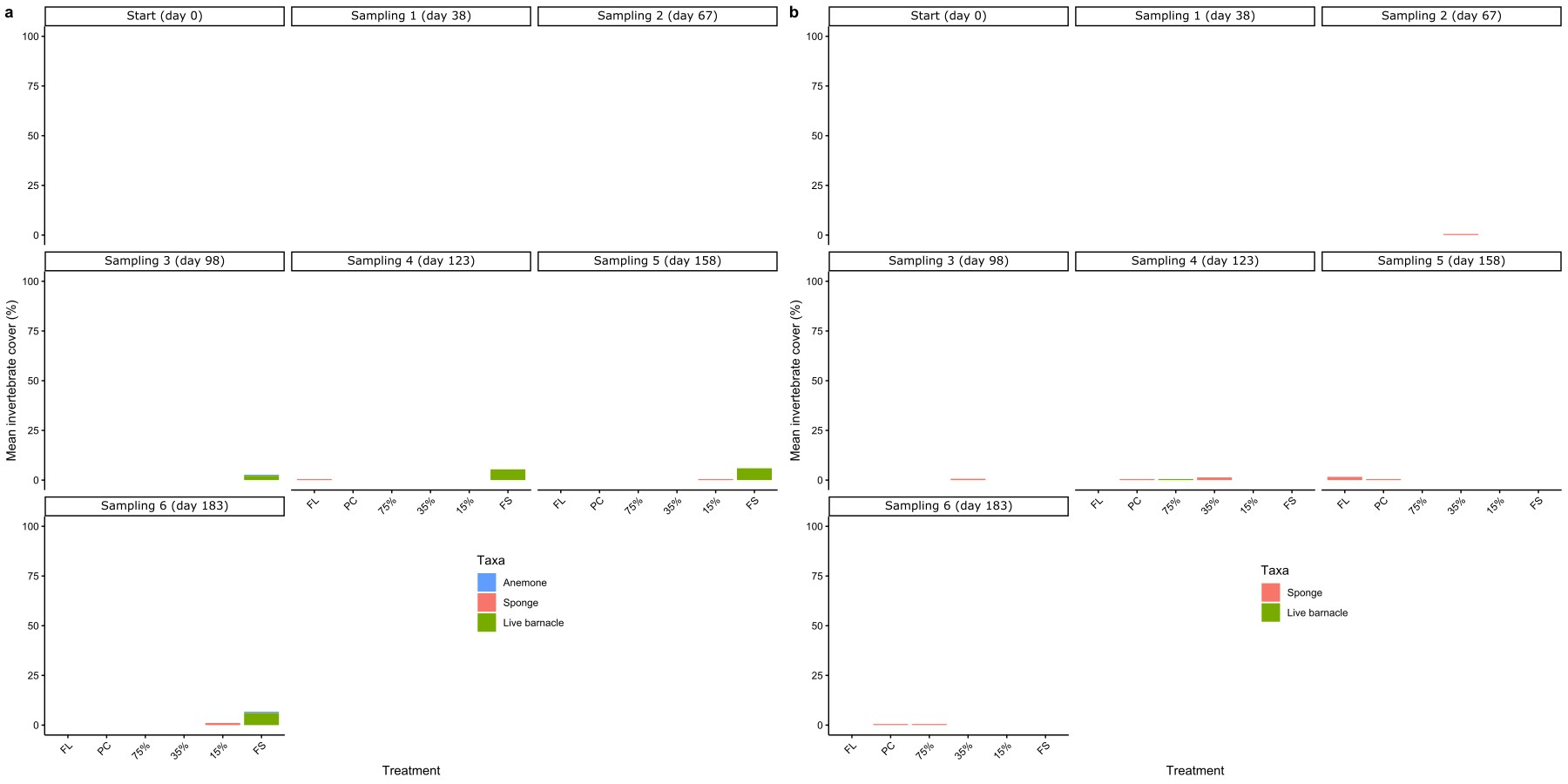
**Fig. S7** Mean cover of sessile taxa besides algae per sampling time at (a) Cape Banks North and at (b) Cape Banks East averaged across replicate pools. FL = Full light, PC = Procedural control, 75% = 75% light transmission, 35% = 35% light transmission, 15% = 15% light transmission, FS = Full shade (see Table S2 for number of replicates per treatment)


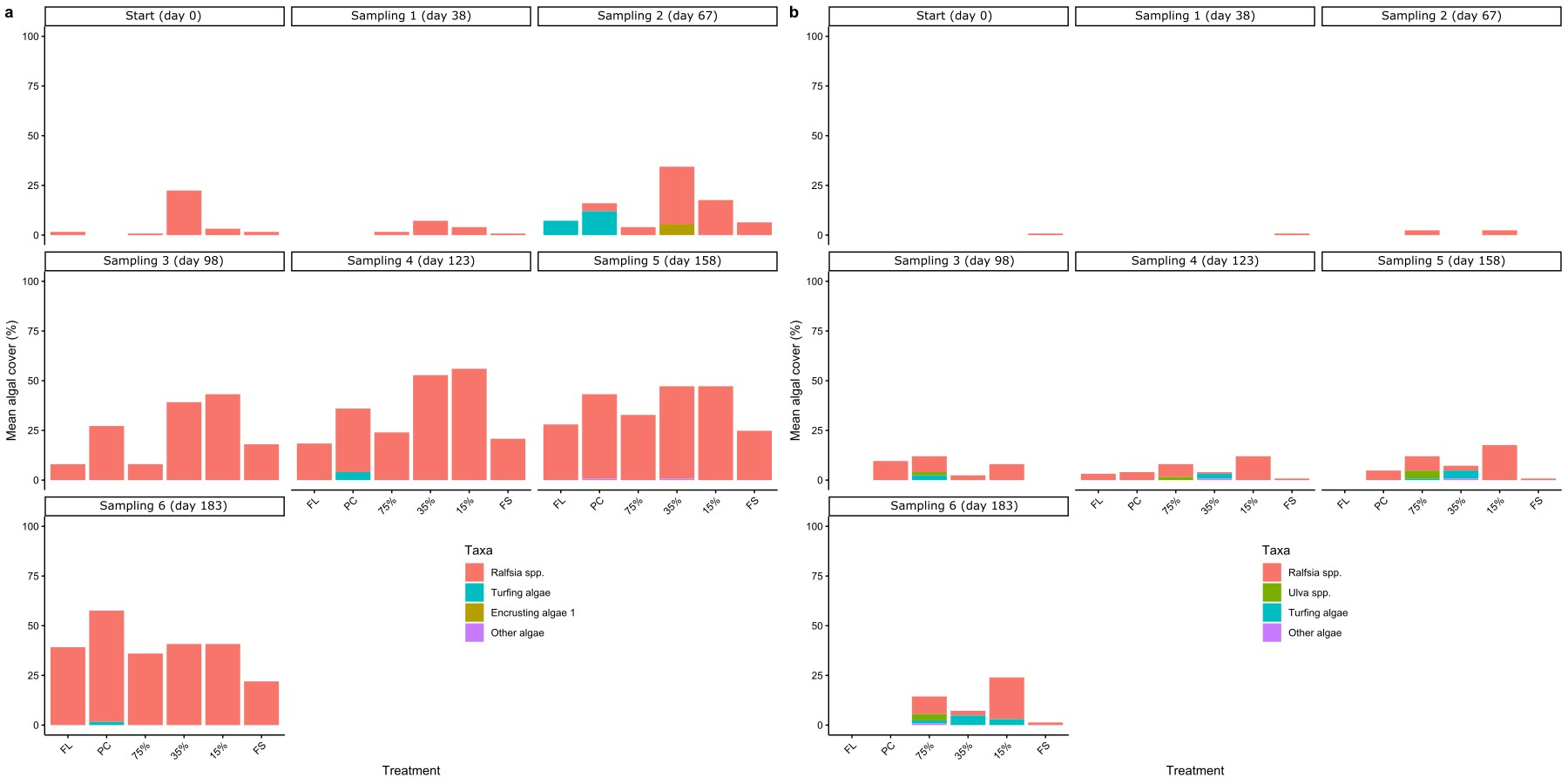


**Fig. S8** Mean algal cover (%) of the three most abundant and other taxa per sampling time at (a) Cape Banks North and at (b) Cape Banks East averaged across replicate plots on emergent rock. FL = Full light, PC = Procedural control, 75% = 75% light transmission, 35% = 35% light transmission, 15% = 15% light transmission, FS = Full shade (see Table S2 for number of replicates per treatment)


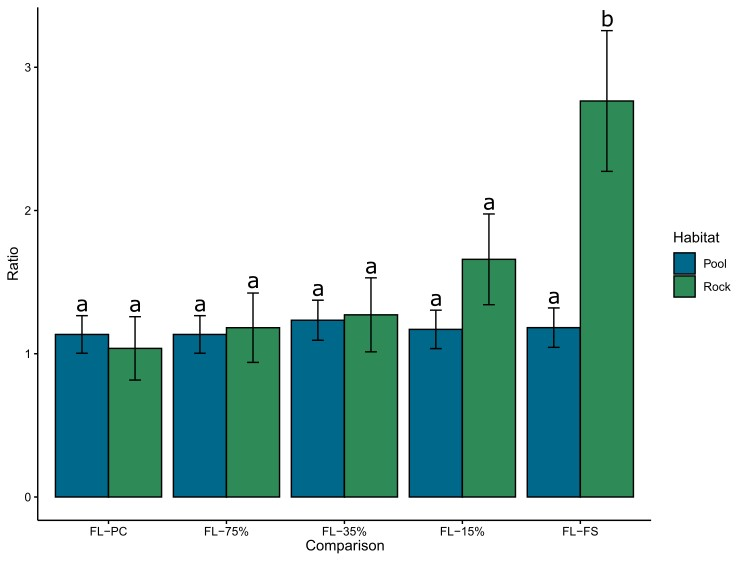


**Fig. S9** Ratio (i.e., effect size) of different light treatments on the richness of mobile taxa compared to the full light treatment for pools and emergent rock at CB North, averaged across time. Error bars are model predicted means and standard errors. Different letters indicate significant differences between habitats for each full light-light treatment comparison (see Table S2 for number of replicates per treatment)


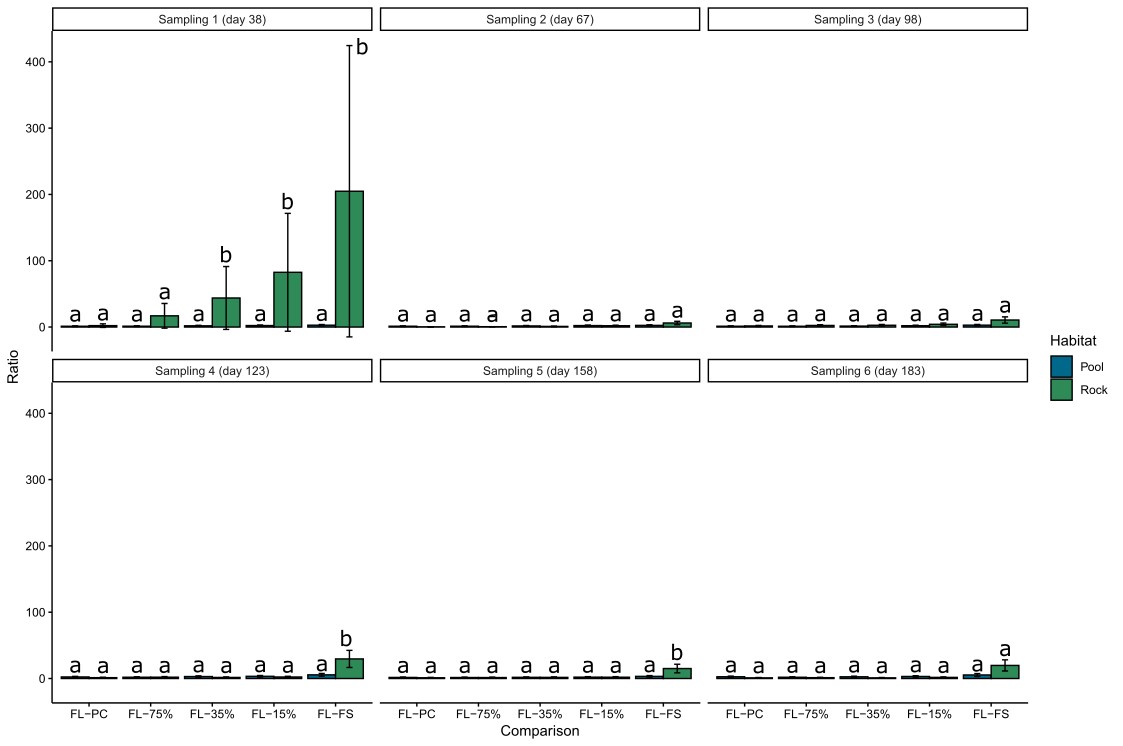


**Fig. S10** Ratio (i.e., effect size) of different light treatments on the abundance of mobile organisms compared to the full light treatment for pools and emergent rock at CB North. Error bars are model predicted means and standard errors. Different letters indicate significant differences between habitats for each full light-light treatment comparison (see Table S2 for number of replicates per treatment)

**Table S7** Presence of sessile taxa under at least one replicate per treatment for each treatment at the start of the experiment and at each sampling. R=*Ralfsia* spp., P=*Petalonia* spp., U=*Ulva* spp., T=Turfing algae, Co=*Corallina* spp., Cp=*Colpomenia* spp., Al= Algae species 1, B=Barnacle, S=Sponge, An=Anemone, Ea1=Encrusting algae 1 FL = Full light, PC = Procedural control, 75% = 75% light transmission, 35% = 35% light transmission, 15% = 15% light transmission, FS = Full shade (see Table S2 for number of replicates per treatment)

| **CB North**  **(rock pool)** | Start  (day 0) | Sampling 1  (day 38) | Sampling 2  (day 67) | Sampling 3  (day 98) | Sampling 4  (day 123) | Sampling 5  (day 158) | Sampling 6  (day 183) |
| --- | --- | --- | --- | --- | --- | --- | --- |
|  |  |  |  |  |  |  |  |
| FL | R | R, T | R, T | R, Co, T | R, U, Co, T, Ea1, S | R, Co, P, T, Ea1 | R, Co, P, T, Ea1 |
| PC | R | R, T | R, U, Cp, T | R, U, Cp, T | R, U, Cp, T, Ea1 | R, U, Co, P, Cp, T | R, U, Co, P, Cp, T, Ea1 |
| 75% | R | R, U, T | R, U, T | R, U, T | R, U, Cp, T, Ea1 | R, U, P, Cp, T | R, U, P, T |
| 35% | R, T | R, U, T | R, U, Cp | R, U, T | R, U, Cp, T | R, U, P, T | R, U, P, T |
| 15% | R | R, Cp, T | R, Cp, T | R, T, Ea1 | R, U, Cp, T, Ea1 | R, U, P, Cp, T, Ea1, S | R, U, P, Cp, T, Ea1, S |
| FS | R, T | R, T | R, Cp, T | R, U, Cp, An, B | R, U, B | R, U, Ea1, B | R, T, An, B |
|  |  |  |  |  |  |  |  |
| **CB North**  **(emergent rock)** | Start  (day 0) | Sampling 1  (day 38) | Sampling 2  (day 67) | Sampling 3  (day 98) | Sampling 4  (day 123) | Sampling 5  (day 158) | Sampling 6  (day 183) |
| FL | R |  | T | R | R | R | R |
| PC |  |  | R, T | R | R, T | R, U | R, T |
| 75% | R | R | R | R | R | R | R |
| 35% | R | R | R, Ge | R | R | R, U | R |
| 15% | R | R | R | R | R | R | R |
| FS | R | R | R | R | R | R | R |

| **CB East**  **(rock pool)** | Start  (day 0) | | Sampling 1  (day 38) | | Sampling 2  (day 67) | | Sampling 3  (day 98) | | Sampling 4  (day 123) | | Sampling 5  (day 158) | | Sampling 6  (day 183) | |
| --- | --- | --- | --- | --- | --- | --- | --- | --- | --- | --- | --- | --- | --- | --- |
| FL | R | | R | | R, T | | R, T | | R, T | | R, U, T, S | | R, U, T | |
| PC | R | | R, T | | R, T | | R, U, T, Ea1 | | R, U, P, T, Ea1, S | | R, U, P, Ea1, S | | R, U, P, Ea1, S | |
| 75% | R | | R, U, T, Al | | R, U, T, Ea1 | | R, U, T, Ea1 | | R, U, Ea1, B | | R, U, Ea1 | | R, U, Ea1, S | |
| 35% | R | | R, T | | R, U, T, S | | R, U, T, S | | R, U, T, S | | R, U | | R, U | |
| 15% | R | | R, T | | R, U, T | | R, U, T | | R, U | | R, U | | R, U | |
| FS | R | | R | | R, U | | R, U | | R | | R | | R | |
|  |  | |  | |  | |  | |  | |  | |  | |
| **CB East**  **(emergent rock)** | | Start  (day 0) | | Sampling 1  (day 38) | | Sampling 2  (day 67) | | Sampling 3  (day 98) | | Sampling 4  (day 123) | | Sampling 5  (day 158) | | Sampling 6  (day 183) |
| FL | |  | |  | |  | |  | | R | |  | |  |
| PC | |  | |  | |  | | R | | R | | R | |  |
| 75% | |  | |  | | R | | R, U, T | | R, U | | R, U, T | | R, U, P, T |
| 35% | |  | |  | |  | | R | | R, P, T | | R, P, T | | R, T |
| 15% | |  | |  | | R | | R | | R | | R | | R, T |
| FS | | R | | R | |  | |  | | R | | R | | R |


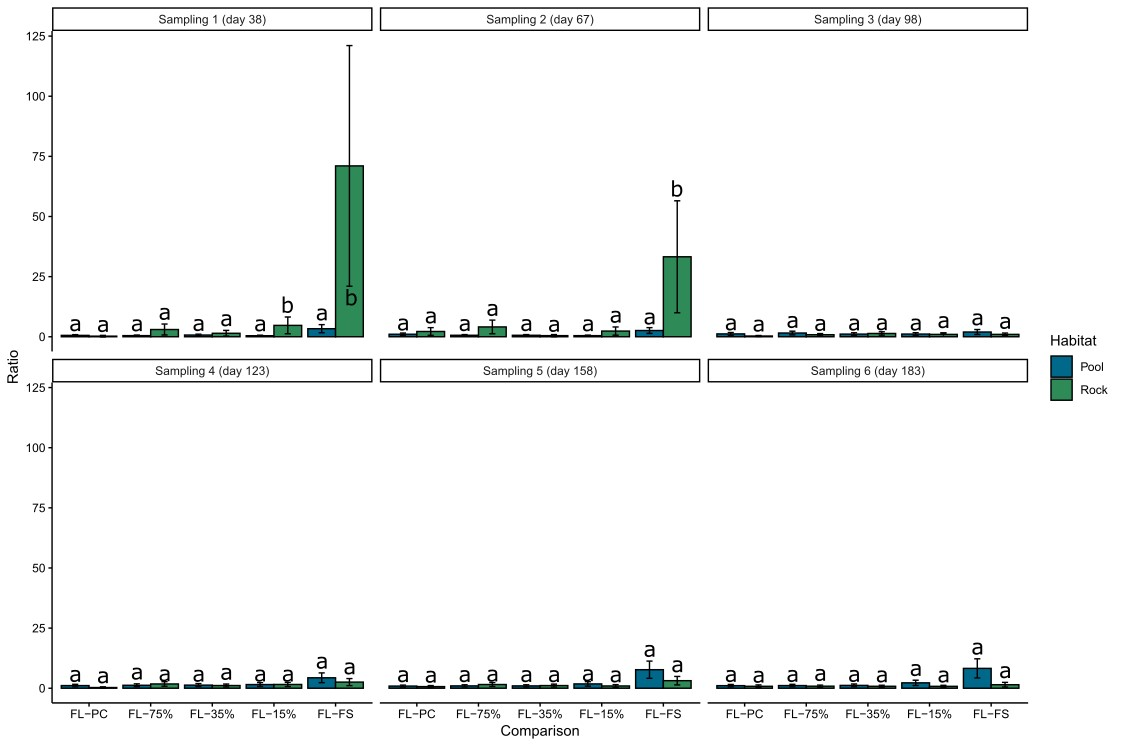


**Fig. S11** Ratio (i.e., effect size) of different light treatments on the abundance of mobile organisms compared to the full light treatment for pools and emergent rock at each sampling time at CB East. Error bars are model predicted means and standard errors. Different letters indicate significant differences between habitats for each full light-light treatment comparison (see Table S2 for number of replicates per treatment)

1. *NS, KD, EL and MMP conceived the ideas and designed the methodology; NS collected the data; NS and GC analysed the data; NS led the writing of the manuscript. All authors contributed critically to the drafts and gave final approval for publication.* [↑](#footnote-ref-1)
